# Global Analysis of the Small RNA Transcriptome in Different Ploidies and Genomic Combinations of a Vertebrate Complex – The *Squalius alburnoides*

**DOI:** 10.1371/journal.pone.0041158

**Published:** 2012-07-18

**Authors:** Angela Inácio, Joana Pinho, Patrícia Matos Pereira, Luca Comai, Maria Manuela Coelho

**Affiliations:** 1 Centro de Biologia Ambiental, Departamento de Biologia Animal da Faculdade de Ciências, Universidade de Lisboa, Lisboa, Portugal; 2 Departamento de Biologia & CESAM, Universidade de Aveiro, Aveiro, Portugal; 3 Department of Plant Biology and Genome Center, University of California Davis, Davis, California, United States of America; Institut Jacques Monod, France

## Abstract

The *Squalius alburnoides* complex (Steindachner) is one of the most intricate hybrid polyploid systems known in vertebrates. In this complex, the constant switch of the genome composition in consecutive generations, very frequently involving a change on the ploidy level, promotes repetitive situations of potential genomic shock. Previously in this complex, it was showed that in response to the increase in genome dosage, triploids hybrids could regulate gene expression to a diploid state. In this work we compared the small RNA profiles in the different genomic compositions interacting in the complex in order to explore the miRNA involvement in gene expression regulation of triploids. Using high-throughput arrays and sequencing technologies we were able to verify that diploid and triploid hybrids shared most of their sequences and their miRNA expression profiles were high correlated. However, an overall view indicates an up-regulation of several miRNAs in triploids and a global miRNA expression in triploids higher than the predicted from an additive model. Those results point to a participation of miRNAs in the cellular functional stability needed when the ploidy change.

## Introduction

**Figure 1:**
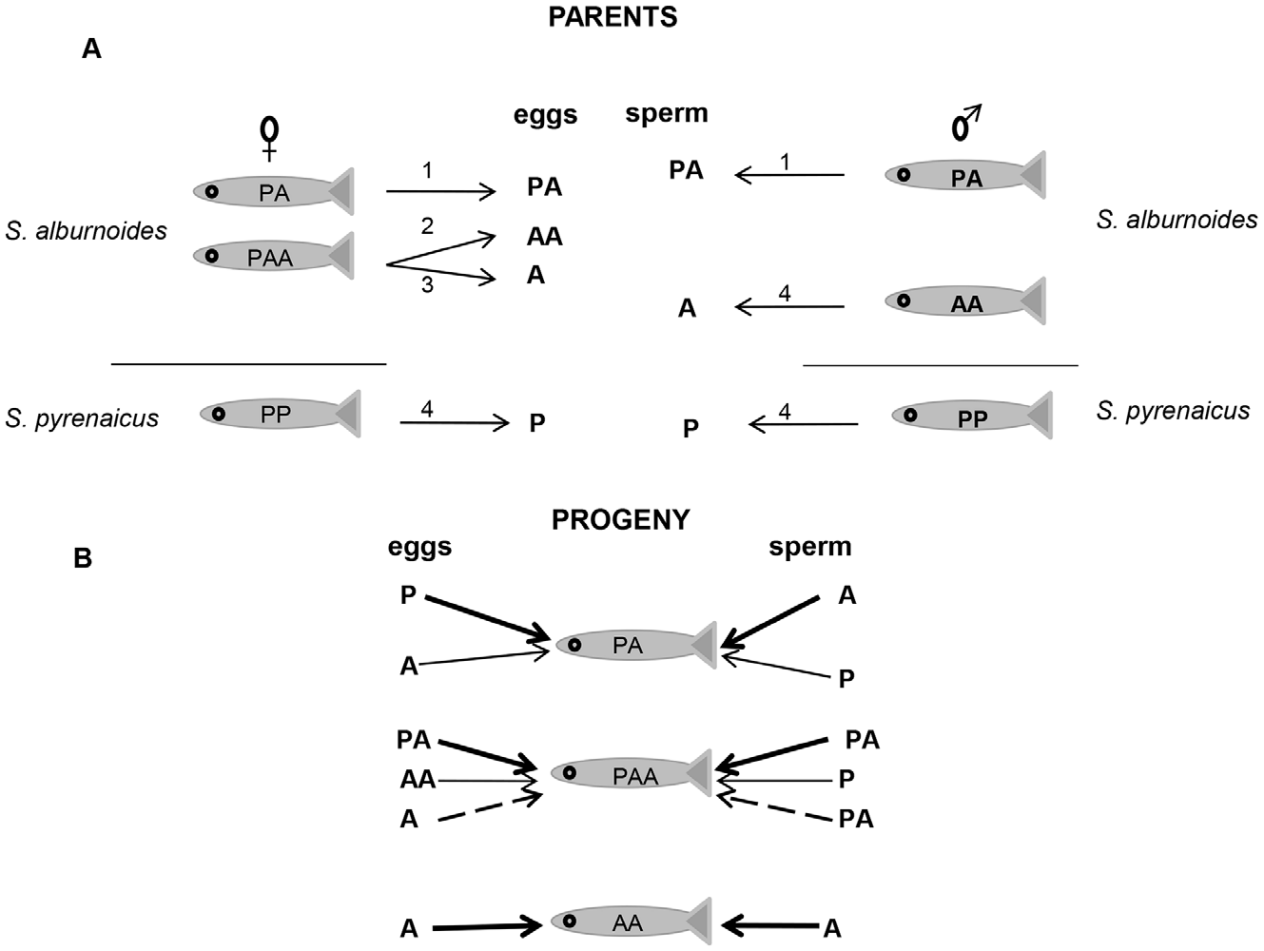
Reproductive mechanisms in the interacting forms of the *S. alburnoides* complex. A) Gametes production - Gametes are produced clonally (1), by hybridogenesis (2), by meiotic hybridogenesis (3) or by normal meiosis (4). AA are always males and PAAs are mainly females. Only the most frequent forms of the complex are presented. B) Possible progenies – PAA, PA and AA can be formed from different gametes combinations. Each combination of gametes is represented by a different type of arrows.

The *Squalius alburnoides* complex (Steindachner) is one of the most intricate hybrid polyploid systems known in vertebrates. This cyprinid fish forms a widely distributed complex endemic to the Iberian Peninsula. It originated by hybridization between a female *Squalius pyrenaicus* (Günther) (P genome) and an already extinct paternal ancestor related to *Anaecypris hispanica* (Steindachner) (A genome) [1]. Nowadays, in the southern basins, the fish is sympatric with the parental species *S. pyrenaicus* (P genome), which still interacts with the complex as a sperm donor and thus acts as source of genetic material [1]. A characteristic feature of the *S. alburnoides* complex is its high reproductive diversity. This promotes intricately networked genetic exchanges and continuous shifting between different genetic forms. Indeed, several mechanisms of sexual and asexual reproduction have been described in this complex [2], [3] and gamete production is assured by hybridogenesis, meiosis and meiotic hybridogenesis. As a result, the complex is composed of hybrids with different ploidies and genomic constitutions, with the most common forms present in southern rivers being the triploids PAA and the diploids PA and AA. AA is extinct as an independent species but is frequently reconstituted from the complex (**Figure 1**).

The frequent changes in genome composition and ploidy entail dosage and hybridity changes that might produce genomic instability. However, the evolutionary success of this complex [1] suggests that it may have developed mechanisms that stabilize its genome. These mechanisms are likely to involve gene expression regulation. Indeed, gene silencing was observed in different tissues of PAA allotriploids by studying allelic regulation of selected house-keeping and tissue-specific genes [4]–[6]. Additionally, mRNA levels of PA diploids resembled those of triploids PAA [4]–[5], suggesting dosage compensation. MicroRNAs (miRNAs), which are involved in a wide range of biological processes, including cell proliferation, differentiation, apoptosis and metabolism [7]–[10], are candidate regulators for the observed silencing and compensation. Computational analysis showed that each animal miRNA can regulate hundreds of different mRNAs, suggesting that a remarkably large proportion of the transcriptome (about 50% in humans) is subjected to miRNA regulation [7], [9]. Involvement of miRNA in hybrid and polyploidy regulation is supported by observations in allopolypoloid plants where genome-wide expression analyses indicate that many genes and miRNAs are expressed non-additively (differently from the mid-parent value) [11]–[14]. Although animal miRNAs were initially reported to repress target translation with little or no influence on mRNA abundance, currently, a role in mRNA degradation is also emerging [15].

Our aim is to explore miRNA involvement in gene expression regulation needed to control the potential genomic shock created by frequent events of hybridization and polyploidization in the *S. alburnoides* complex. Here, we used specimens from the southern populations of Portugal to compare miRNA transcriptome profiles in different genomic compositions: PAA, PA, AA and PP. Consistent with the previously proposed dosage compensation in the triploid PAA gene expression [4], [5], here we found that small RNA expression profiles in PA and PAA are similar. However a shift in the global miRNA expression was detected between the two, suggesting miRNA involvement in the maintenance of triploid stability.

## Results

### Primary Analysis of the Small RNA Libraries

**Figure 2:**
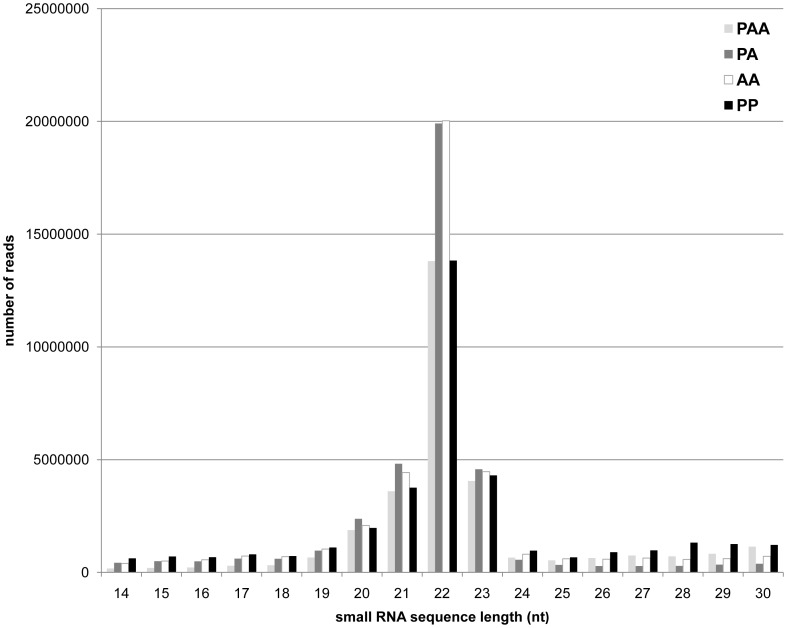
Length distribution of reads in *S. alburnoides* (forms PAA, PA, AA) and *S. pyrenaicus* (PP). Small RNAs are ranged in size from 14 to 30 nucleotides. The prominent peaks at 22 nucleotides confirmed the high quality of small RNA libraries used in the sequencing run.

**Figure 3:**
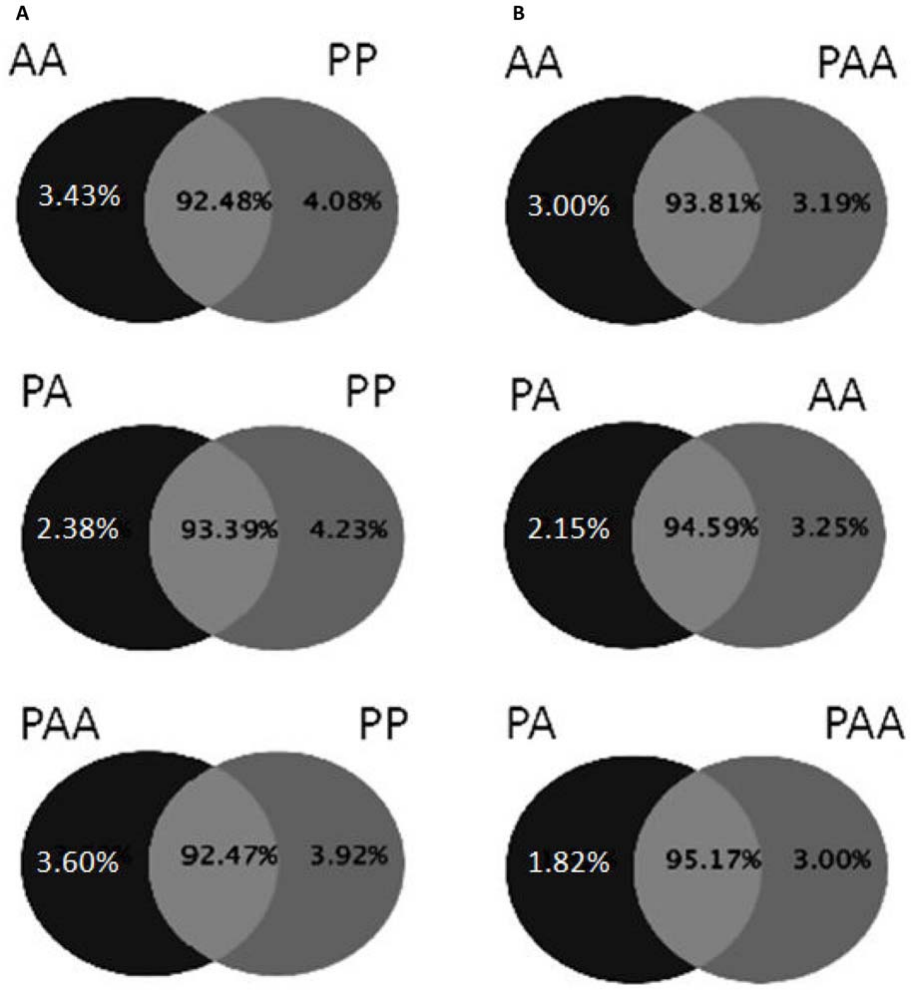
Pairwise analysis of the common and exclusive clean read sequences between libraries. For each library a quantitative analysis of the exclusive (in black or dark grey) and common (light grey) sequences is presented by pairwise comparison with all the other libraries. A) pairwise comparisons between *S. alburnoides* forms (AA, PA and PAA) and *S. pyrenaicus* (PP). B) Pairwise comparisons between the different forms of the complex.

In order to investigate small RNAs transcriptome divergence among the most frequent forms interacting in the *S. alburnoides* complex, RNA was purified from selected tissues, pooled and used for small RNA sequencing library construction in PAA, PA, AA and PP.The selected tissues, liver, muscle and brain, provide good representation of developmental diversity. While liver and muscle showed high levels of gene silencing in triploids [4], brain tissue has been showed to display high diversity of miRNAs in fish [16]. Sequence reads were produced on Illumina GAII sequencers, filtered using quality criteria (see methods) yielding 43.5 M for PAA (65.49% out of the total reads), 43.2 M for PA (61.85%), 44.4 M reads for AA (64.39%), and 42.5 M for PP (60.71%) corresponding to 2.4 M, 1.7 M, 2.1 M and 2.6 M respectively, of unique non-redundant sequences. The length distribution of the reads showed a similar pattern among the four libraries: a distinct peak composed by 20–23 nucleotides in length, the most prevalent length being 22 nucleotides (**Figure 2**). The distribution obtained by sequencing corroborates the libraries quality since the expected length of miRNAs is 20–23 nucleotides [17]. By pairwise comparison, we assessed the presence of library-specific sequences. The majority of sequences were shared by all forms of the complex (mean 94.52%, +/−0.9%) with PA and PAA being the most similar libraries (95.17% of common sequences). *S. pyrenaicus* displayed a slightly more distant profile from the complex (mean 92.78%, +/−0.5%) **(** **Figure 3** **)**, but no statistical significance was found.

### Genome Mapping and Annotation of the Small RNAs Libraries

Given that no sequenced genome for *S. alburnoides* or *S. pyrenaiucus* is available we used another cyprinid, zebrafish (*Danio rerio*), as reference. The quality-filtered reads were mapped to the *Danio rerio* genome resulting in 22 612 648 mapped reads for PAA (52.03%), 28 774 125 reads for PA (66.70%), 29 869 369 for AA (67.30% of total reads) and 24 344 581 for PP (57.21%). The percentages of mapped reads were consistent with the phylogenetic relationship between species belonging to the same family (Cyprinidae) and are in accordance with other studies involving those species [18], [19]. The annotated read tags were divided into different categories of RNAs. Although several classes of RNAs were identified, such as snRNAs, snoRNAs, rRNAs, tRNAs, mRNAs and mRNAs precursors (most probably corresponding to putative degradation products), the major fraction corresponds to miRNAs, with a total of 204 conserved miRNAs identified in all the libraries (**Table S1**). Not surprisingly given the use of another genome as a reference, a considerable fraction of tags corresponded to unannotated sequences. Reads counts and distribution by categories are summarized for each library in **Table S2**.

### Correlation Analysis of miRNA Expression Profiles

**Figure 4:**
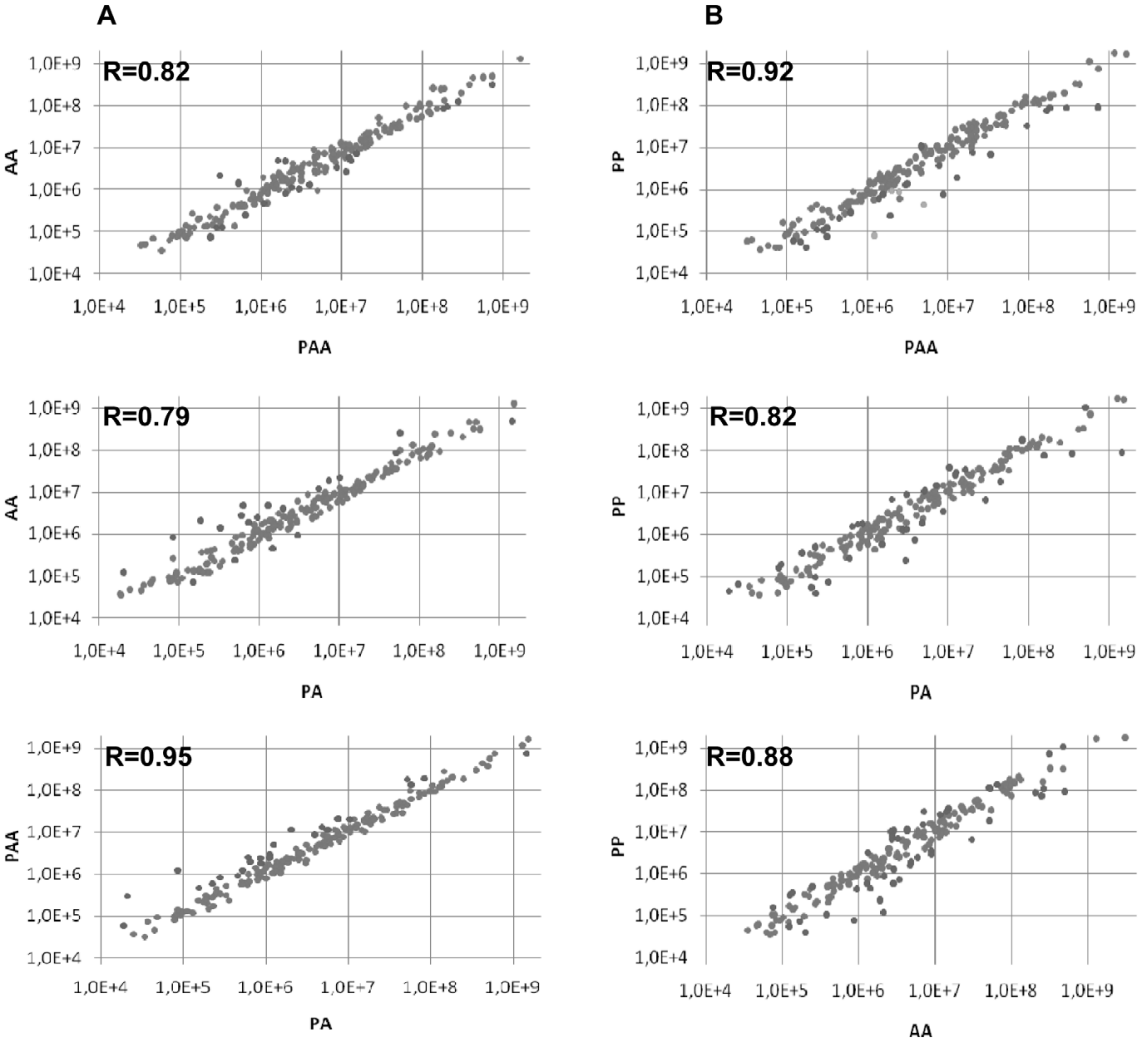
Pairwise comparison of miRNA gene expression profiles obtained by small RNAseq. **A) within** ***S. alburnoides*** **complex B) with the parental species** ***S. pyrenaicus*** **(PP)** The expression of each miRNA was calculated by dividing the number of reads for each miRNA by the total number of miRNAs in the same library. Each dot(•) represents the ratio between the expression of a miRNA in the two genomic compositions. The degree of the relationship between two miRNA profiles is represented by the correlation value (R).

**Figure 5:**
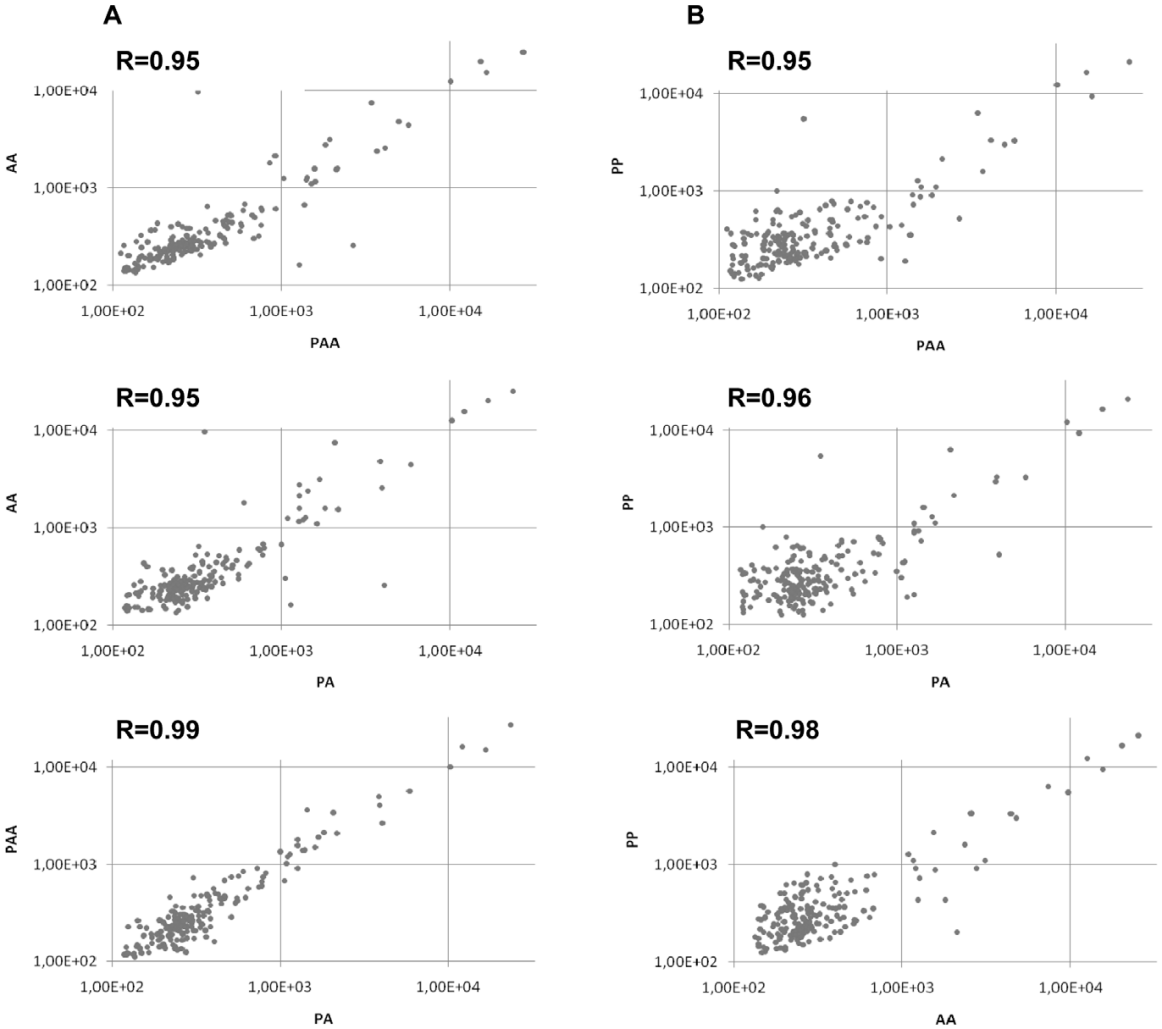
Pairwise comparison of miRNA gene expression profiles obtained by microarrays. **A) within** ***S. alburnoides*** **complex B) with the parental species** ***S. pyrenaicus*** **(PP).** Each dot (•) represents the ratio between the expressions of a miRNA in the two genomic compositions. The degree of the relationship between two miRNA profiles is represented by the correlation values (R).

In order to compare miRNA expression profiles between the different genomic compositions, we made pairwise comparisons and correlations were estimated. Results show that although all the expression profiles were positively correlated (mean r = 0.86, +/−0.06) the most correlated were the PA and PAA profiles (r = 0.95) (**Figure 4**). We then made an analysis of comparable design by hybridizing cDNA to microarrays containing a set of *Danio rerio* miRNA probes (**Table S1**). Again, the best correlation was between PA and PAA (**Figure 5**).

### Comparison of the Global miRNA Expression Analysis between the Interacting Forms

**Figure 6:**
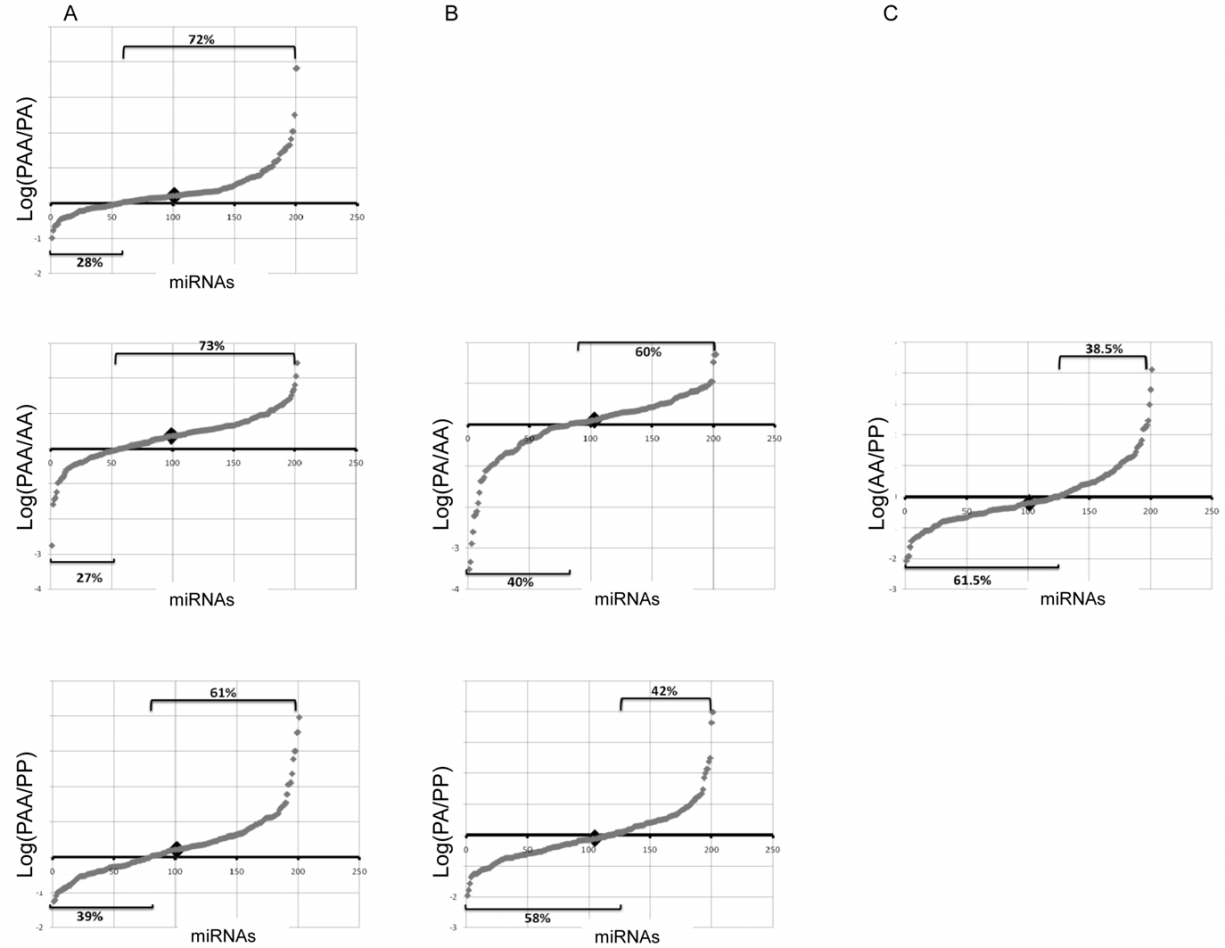
Differences in global miRNA expression profile. Differences related to **A**) allopolyplodization and **B**) hybridization. **C**) Differential expression profile between the parental species. Logarithmized ratios of gene expression for each miRNA were plotted producing a characteristic crescent curve where positive values indicate miRNAs with a higher expression and negative values miRNAs with a lower expression. ♦ Represents the median and its position indicate if most of the values are positive or negative. The percentages of positive and negative values are indicated.

Afterwards, we decided to analyze whether the global expression profile of miRNAs changes with ploidy increase in such a way that, by compensating the gene dosage, could enhance stability of the triploids. No major differences of miRNA expression levels were found in any of the possible comparisons, either in the microarrays or in the small RNAseq analysis. Indeed, the majority of fold changes were between 0.5 to 2 (**Figure S1**). Therefore, we focused on the moderate expression changes detected in the small RNAseq data, deciding that the lower sensitivity of microarrays and the decreased fidelity of *Danio rerio* miRNA chip could compromise accuracy. For the analysis, normalized counts of each miRNA in one library were divided by the corresponding expression value of the same miRNA in the other library; log ratios were plotted producing a characteristic crescent curve where positive values indicate miRNAs with a higher expression and negative values mRNAs with a lower expression (**Figure 6**). Results indicated that the increase in the ploidy level affected the miRNA transcriptome, since the analysis between diploids and triploid hybrids showed a significant difference in the number of up and down-expressed miRNAs (χ-test, p<0.001), with an higher amount of up-regulated miRNAs in triploids (72%) (**Figure 6A**). However, no conclusions about the absolute up-regulation in block of all miRNAs in PAAs can be made, since the same molar amount of small RNA library was used for sequencing. Nevertheless, small RNAseq reveals a change in the global profile of PAAs, with a higher percentage of miRNAs being up-regulated in comparison to any other diploid (**Figure 6A**). In support of this evidence, the majority of up-regulated miRNAs (93%) in PAA/PA were also up-regulated in at least one of the other comparisons (PAA/PP or PAA/AA) and 62% were up-regulated in all the three comparisons (**Table S3**). If we considered only the miRNAs that change their expression more than 1 fold, 21% are up-regulated in all the comparisons and correspond to 5 miRNAs: dre-mir-182, dre-mir-183, dre-mir-96, dre-mir-141 and dre-mir-192 (**Table S3**). No specific transcript or group of transcripts sharing the same gene ontology term was found to be targeted by all the 5 miRNAs (MicroCosm Targets Version 5 and [19]).

Hybridization does not seem to cause a major impact in the relative expression profile of miRNAs, since the number of up and down-expressed miRNAs is not dramatically different between the PA hybrid and any of the others non-hybrid diploids (**Figure 6B**). Indeed, the relative differences in the miRNA expression profiles between hybrids and non-hybrids diploids seem to be related with the differences in the miRNA expression profiles between the non-hybrid forms PP and AA (**Figure 6C**). In agreement with that, 80% of the up or down-regulated miRNAs in PA/PP and 72% of the up or down-regulated miRNAs in PA/AA changed their expression in a consistent direction with the AA/PP comparison (**Table S3**), with only 4 miRNAs expressing differently in both comparisons (PA*/*PP and PA/AA) from the expected from the AA/PP tendency. However, these 4 miRNAs (dre-mir-727, dre-mir-27d, dre-mir-27b, dre-mir-142b-5p) show moderate changes, meaning less than 1 fold, in any comparison (PA/PP, PA/AA and PP/AA)(**Table S3**).

**Figure 7:**
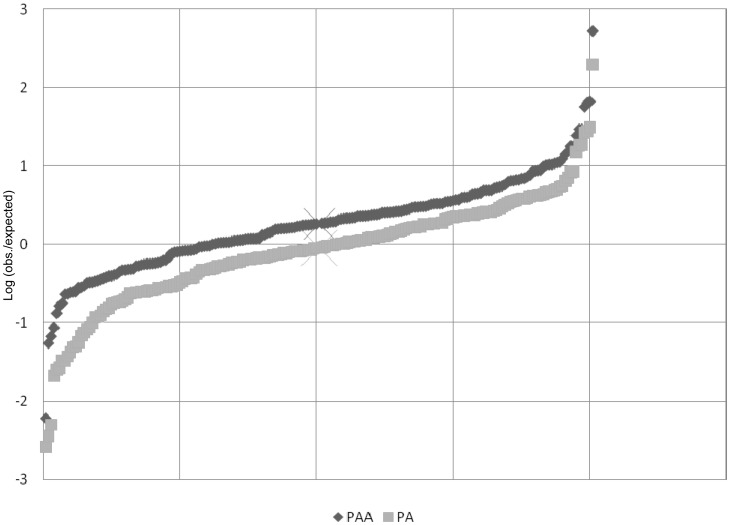
Additivity in miRNA expression in PAA and PA. Additivity for each miRNA was calculated by dividing the observed value by the expected value. The expected value was obtained using the miRNA expression values of the non-hybrid diploids - AA and PP (the expected value for PA is (PP+AA)/2 and the expected value for PAA is (PP+2AA)/3)). For PAA and PA, the logarithmized ratios for each miRNA were plotted producing a characteristic crescent curve where positive values indicate miRNAs with a higher expression and negative values miRNAs with a lower expression than expected. (X) Represents the median and its position indicate if most of the values are positive or negative.

The additivity in miRNA expression in the hybrid forms was also investigated. For that, we used the miRNA expression levels of the non-hybrid diploids (AA and PP) to calculate the expected expression. To satisfy the expectation of additivity, PA expression should equal (PP+AA)/2 and PAA should equal (PP+AA+AA)/3). In both cases, the real values of expression do not differ markedly from the expected values (−1<log(real/expected)<1 in 89% of the miRNAs; **Table S4**). However, while in PA we observed nearly the same amount of up or down-expressed miRNAs in relation to the additive expectation (median–0.043; χ-test, p = 0.624), indicating small fluctuation around zero (no difference), in PAA there is a higher number of the over-expressed miRNAs comparing to the down-expressed ones (70% *vs* 30%)(median−0.26; χ-test, p<0.01) (**Figure 7**). This analysis confirmed that miRNAs expression in the triploids deviates from additivity, consistent with adjustments that may maintain the functionality of the triploid cell.

## Discussion

The frequent formation of ploidy and genomic composition variations in the in the *S. alburnoides* complex (**Figure 1**) should promote repetitive situations of genomic imbalance and instability. However, this hybridogenetic complex shows high evolutionary success, and so it is an excellent organism to investigate allopolyploidization [1], [3]. The allopolyploid *S. alburnoides* complex must overcome these genetic constrains in order to achieve a functional gene expression. The ability to reach phenotypic homeostasis is demonstrated by comparison of diploids (PA) and triploids (PAA), which are morphologically undistinguishable (PP and AA are easily distinguishable) [20]. This suggests that gene expression in PAA is non-additive. The presence in triploid vertebrates of a compensating silencing mechanism was first suggested by Pala *et al*. [4]. Consistent with this proposal, the increase of A gene dosage in a triploid was compensated since the PAA reduced transcript levels to that of the PA diploid. This phenomenon was shown to be persistent in several river habitats where *S. pyrenaicus* is sympatric with *S. alburnoides* [6].

Deviation for additivity has been reported for polyploidy plants [21]. For instance, a study from Guo [22] described dosage-dependent (additive) regulation for several maize genes, and dosage compensation for a few genes. Many miRNA and their targets were not additively expressed in *Arabidopsis* allotetraploids [13], [14], suggesting a miRNA’s functional role in gene regulation. MicroRNAs, which are post-transcriptional gene expression modulators play major roles in several biological processes in plants and animals and are prime candidates to alleviate the genetic constrains generated in polyploid organisms. However, while a miRNAs involvement in non additive gene regulation has been reported in allopolyploid plants [23], a genome-wide role in dosage compensation has not yet been documented.

In this study, miRNA expression profiles of the three most frequent forms of *S. alburnoides* (genomic compositions AA, PA and PAA), as well as the parental species *S. pyrenaicus* (PP) were assessed using next generation sequencing and microarrays hybridization. In the first approach, the Illumina platform provided deep coverage of the small RNA transcriptome. About 60% of quality-filtered reads could be aligned to the *Danio rerio* genome, confirming the high conservation level between *Squalius pyrenaicus* and *Squalius alburnoides* with the *Danio* reference genome [18], [19]. Additionally, the length distribution of the reads indicated predominant representation of 22 nucleotides, the expected size for miRNAs (**Figure** **2**). Although our results do not show similar number of reads with 22 nts for all the libraries, this probably reflects their qualities, since libraries from PP and PAA appeared less concentrated during gel electrophoresis analysis. This can also explain why PP and PAA display more reads in 25–30 lengths: since the lower amount of 22 nts reads generates a relative increase in the reads of different size classes. After the genome alignment, sequences were annotated with several databases revealing a set of 204 conserved miRNAs. The miRNAs proportion in the libraries was in accordance with other studies [24]. So, given the satisfactory quality of all libraries’ parameters, further analysis was performed. Before mapping or annotation, specific and common reads were assessed in each library. The libraries that presented more common sequences were PA and PAA, which share genomes P and A. This work shows that also in the small transcriptome, the increase of the gene dosage in *S. alburnoides* does not seem to promote a big change, since PA/PAA pairwise comparison are the most similar profiles in terms of sequences being expressed, reflecting the more or less random inactivation of genomic-specific alleles (A or P) in PAAs [6]. The high similarity in the small transcriptomes between the two nuclear hybrid forms (PA and PAA) was also demonstrated by pairwise comparison of their expression profiles, which were the most correlated of all the possible pairwise comparisons both in the sequencing and arrays analyses (**Figures 4** **and** **5**). The good correlation in the global miRNA expression between PA and PAA is consistent with a convergent adaptation to similar mRNA environments, supporting once more a compensatory dosage effect in triploids. Indeed, even if there is a shift in the global miRNA expression in one of the forms, the high correlation shows that the fold changes are occurring in the same direction for the majority of miRNAs.

In order to study modest fold changes in the global miRNA expression, we focused on the small RNAseq data, which has been shown to yield more reliable absolute quantitative measurements and a better approximation of the real transcript content than microarrays [25]–[28]. Microarrays cannot easily discriminate transcripts from repeated sequences nor subtle changes in gene expression levels [25]–[28]. In the comparison of PAA to PA, small RNAseq analysis detected a significant difference in the number of up-regulated miRNAs compared with the number of down-regulated (**Figure 6A**). Surprisingly, triploids displayed more up-expressed miRNAs than any other diploid genome composition. Besides, the majority of up-regulated miRNAs in PAA/PA were also up-regulated in the other triploid/diploid comparisons (PAA/PP and PAA/AA). If we apply a cutoff of 1 fold change to all the triploid/diploid comparisons, 5 miRNAs were consistently over-expressed: mir-182, mir-183, mir-96, mir-141 and mir-192. However, no particular transcript or group of transcripts sharing the same gene ontology is targeted by all the 5 miRNAs. We concluded that miRNA up-regulation is a generalized response of the genomic shock created by the ploidy increase and no particular pathway is affected. Additivity analysis confirmed the up-regulation scenario, as the expression levels of several miRNAs were higher than expected from genome composition. This is consistent with discovery in plants where Ha *et al.* [29] reported deviations from the mid-parental value in miRNAs expressed in a newly made allotetraploid hybrid homologous to *Arabidopsis suecica*. More recently, Kenan-Eichler *et al.* [30] found that in wheat the relative amount of miRNAs increases with ploidy level. Consistent with our findings, the wheat study showed that while in most diploid hybrid miRNA expression profiles did not deviate from their mid-parent value, a deviation occur in the allopolyploid.

Our results reveal two responses, a dosage compensatory one acting in PAA resulting in a small RNA expression profile similar to PA, and a genome wide regulatory response by miRNAs that although moderate could play an important role in the maintenance of a diploid mRNA expression level in the triploids. The last is probably promoting genome stability contributing to the evolutionary success of this allopolyploid complex.

## Materials and Methods

### Ethics Statement

Electric fishing was performed by Inácio A under her authorization from Autoridade Florestal National (from by the Ministry of Agriculture and Fishery in Portugal) - Credencial de Pesca n° 22/2011. Other colleagues with licenses also participated in fishing (Credencial de Pesca numbers 25/2011, 26/2011, 29/2011). A named review board institution - Instituto da Conservação da Natureza e da Biodiversidade, I.P (from the Ministry of Environment in Portugal) approved the fish handling and sacrifice (licenses numbers 179/2011/CAPT, 180/2011/CAPT, 181/2011/CAPT). Our institution also has the authorization from Direção-Geral de Veterinária (from the Ministry of Agriculture) to use animals for scientific experiments (Ofício circular n° 99 042/000/000 9/11/2009). In this work, animals were never distressed and overdose of anaesthesia tricaine mesylate (MS-222) was used for the fish sacrifice. All the procedures followed the recommendations from Federation of Laboratory Animal Science Associations.

### Sampling and Genomic Constitution Determination

*S. alburnoides* specimens were collected from Almargem River and *Squalius pyrenaicus* from Colares. Fish were captured by electrofishing and presented approximately 10 cm long. Each genome contribution was determined according to Inácio *et al.* [31].

### RNA Extraction

In order to ensure that miRNA expression was not affected by external factors such as stress, fish were acclimated in captivity for two weeks. Afterwards, they were sacrificed by overdose on anaesthetic MS222 and the respective tissues/organs dissected and preserved in RNAlatter® (Ambion) at −20°C.Total RNA was extracted from liver, muscle and brain using the Tri-Reagent® (Ambion) and following the suppliers’ instructions. Contaminant DNA was eliminated by the addition of TURBO™ DNase (Ambion) followed by purification with phenol/chloroform. Ethanol, Glycogen and Sodium Acetate (NaOAc) were used to achieve RNA precipitation. Quantity and quality evaluation of the extracted RNA was performed in Nanodrop 1000 (Thermo Scientific) and in 2100 Bioanalyser (Agilent Technologies). The concentrations were also registered.

### Library Construction and Sequencing Analysis

To construct the smallRNA libraries, only RNA samples with a RIN higher than 8 (Bioanalyser) were considered. Three different organs (muscle, brain and liver) from an average of three individuals of the same genomic composition were pooled together for library construction. Four libraries were made: 1 for *S. pyrenaicus*- PP and 3 for *S. alburnoides-* AA, PA and PAA. Each library was made following the Illumina protocol *Small RNA v1.5*. In this case small RNAs were isolated by size in a Polyacrylamide Gel Electrophoresis, followed by band excision. Libraries were shipped on dry ice to Beijing Genomics Institute (BGI), Hong Kong, where they passed the sample quality requirements for sequencing: Qubit Fluorometer, Agarose Gel Electrophoresis, and Agilent 2100 for concentration; Agilent 2100 for fragment size; Q-PCR for molar concentration. The same amount of each library was sequenced by the Illumina technology. Reads were assembled and annotated using *Danio rerio* as reference. Small RNA tags were mapped to the genome by SOAP using the Ensembl database (ftp://ftp.ensembl.org/pub/release-64/fasta/danio_rerio/dna/) and annotation was made not only using the miRBase database (http://www.mirbase.org/) for the precursor/mature miRNAs but also the GenBank (http://www.ncbi.nlm.nih.gov/) and Rfam (http://www.sanger.ac.uk/software/Rfam) databases, since many other sequences from exons, introns, tRNA, rRNAs, snoRNAs and snRNA were presented. All the clean tags were grouped so that each unique sequence from each category had its associated number of reads (counts). To ensure that every unique small RNA sequence was mapped to only one annotation, the following priority rule was used: rRNA, etc (in which Genbank > Rfam) > known miRNA > repeat > exon > intron [32]. Each miRNA expression level was calculated by dividing the number of reads of that miRNA by the total reads of all miRNAs in the same library. The data discussed in this publication have been deposited in NCBI’s Gene Expression Omnibus [33] and are accessible through GEO Series accession number GSE38691 (http://www.ncbi.nlm.nih.gov/geo/query/acc.cgi?acc=GSE38691). Gene ontologies were analyzed by DAVID Bioinformatics Resources 6.7 (http://david.abcc.ncifcrf.gov/; [34]) using the targets listed in 20. Another analysis by MicroCosm Targets Version 5 (http://www.ebi.ac.uk/enright-srv/microcosm/htdocs/targets/v5/) was made in order to confirm the first results, in this case a cutoff of p<1×10^6^ was applied.

### Hybridization in miRNA Microarray and Data Treatment

The same RNA pools that were used to construct the sequencing libraries were also subject to microarray analysis. For that, total RNA was labeled using the ULS microRNA labeling kit (Kreatech). Briefly, two µg of total RNA were incubated with Cy3-ULS (1 µl of Cy-ULS for 1 µg of cDNA) for 15 min at 85°C. The labeled RNAs were purified to remove non-reacted Cy3-ULS to produce a fluorescently-labeled RNA sample for microarray analysis. Dye incorporation was monitored by UV-visible spectroscopy. Hybridization was carried out in the miRNAChip_MS_V1 [35] at 42°C for 16 h. Slides were washed following the manufacture’s recommendations and immediately scanned using an Agilent G2565AA microarray scanner. Microarray images were analyzed using QuantArray v3.0 software (PerkinElmer). Cy3 median pixel intensity values were background subtracted, normalized and subject to further analysis. Data points were removed when intensity values were below 200% of background. The normalization of microarray data was applied using BRB-ArrayTool v3.4.0 software. The data discussed in this publication have been deposited in NCBI’s Gene Expression Omnibus [33] and are accessible through GEO Series accession number: GSE36894 (http://www.ncbi.nlm.nih.gov/geo/query/acc.cgi?acc=GSE36894).

### Statistical Analysis

When appropriate, statistical analysis of the data was performed applying the χ-square test or the correlations coefficients. Correlations were calculated using the covariance of the samples and the standard deviations of each sample.

## Supporting Information

Figure S1 **MicroRNAs grouped according to their differential expression in microarrays and smallRNAseq**. Differences in the expression for each miRNA in two different libraries were analyzed by ratios of expression. Fold differences were grouped according to their values (<0.5; >2 or between).The majority of miRNAs shows a fold differences between 0.5 and 2.
(TIF)

Table S1 **Conserved miRNAs annotated in** ***S. alburnoides*** **and** ***S. pyrenaicus*** **from the small RNA data and their presence in the microarray miRNA chip.**
(DOCX)

Table S2 **Annotation of small RNA tags.**
(DOCX)

Table S3 **Differences in the expression levels of each miRNA for all the possible pairwise comparisons – PAA/PA, PAA/PP, PAA/AA, AA/PP, PA/PP, PA/AA.** Fold differences are logarithmized and presented in an ascending order. In grey background are values higher than 1 and lower than −1.
(DOCX)

Table S4 **Differences between real expression values and expected values in case of additivity.** Results are logarithmized log_2_ (real/expected)) and presented in an ascending order. In grey background are values higher than 1 and lower than −1.
(DOCX)
