# Supplementary figures and images for: Global Analysis of the Small RNA Transcriptome in Different Ploidies and Genomic Combinations of a Vertebrate Complex – The Squalius alburnoides
Source: PLoS One. 2012 Jul 18;7(7):e41158. doi: 10.1371/journal.pone.0041158 (PMC3399795; doi:10.1371/journal.pone.0041158)

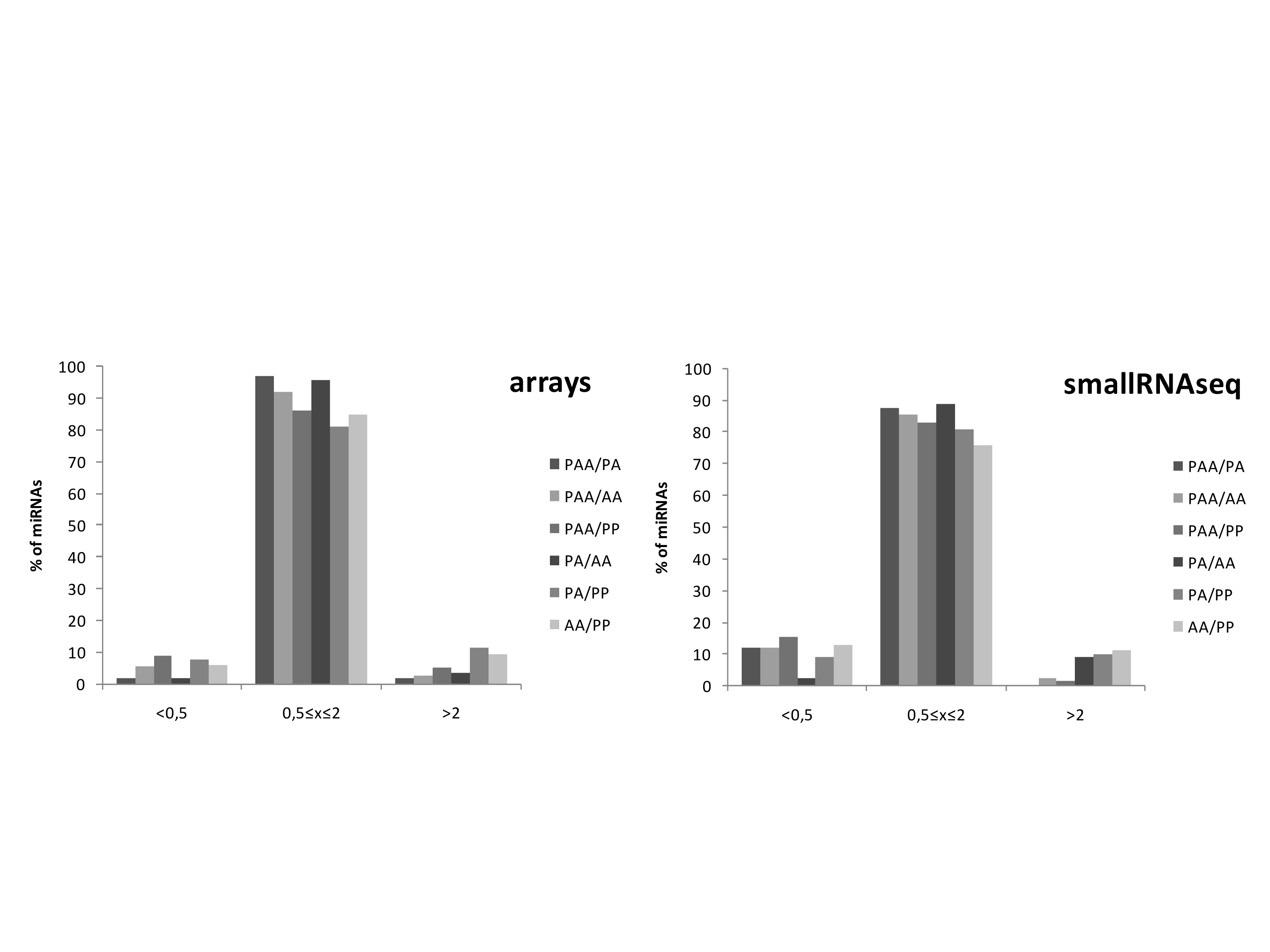

Supplement: Figure S1 — MicroRNAs grouped according to their differential expression in microarrays and smallRNAseq. Differences in the expression for each miRNA in two different libraries were analyzed by ratios of expression. Fold differences were grouped according to their values (<0.5; >2 or between).The majority of miRNAs shows a fold differences between 0.5 and 2. (TIF) [file pone.0041158.s001.tif]
