# Supplementary material for: Global Analysis of the Small RNA Transcriptome in Different Ploidies and Genomic Combinations of a Vertebrate Complex – The Squalius alburnoides
Source: PLoS One. 2012 Jul 18;7(7):e41158. doi: 10.1371/journal.pone.0041158 (PMC3399795; doi:10.1371/journal.pone.0041158)
Supplement: Table S1 — Conserved miRNAs annotated in S. alburnoides and S. pyrenaicus from the small RNA data and their presence in the microarray miRNA chip. (DOCX) [file pone.0041158.s002.docx]

| **miRNAs identified by small RNAseq** | **presence in microarrays** |
| --- | --- |
| dre-let-7a | yes |
| dre-let-7b | yes |
| dre-let-7c | yes |
| dre-let-7d | yes |
| dre-let-7e | yes |
| dre-let-7f | yes |
| dre-let-7g | yes |
| dre-let-7h | yes |
| dre-let-7i | yes |
| dre-let-7j | yes |
| dre-miR-1 | yes |
| dre-miR-100 | yes |
| dre-miR-101a | yes |
| dre-miR-101b | yes |
| dre-miR-103 | yes |
| dre-miR-107 | yes |
| dre-miR-107b |  |
| dre-miR-10a | yes |
| dre-miR-10b | yes |
| dre-miR-10c | yes |
| dre-miR-10d | yes |
| dre-miR-122 | yes |
| dre-miR-124 | yes |
| dre-miR-125a | yes |
| dre-miR-125b | yes |
| dre-miR-125c | yes |
| dre-miR-126 | yes |
| dre-miR-126* | yes |
| dre-miR-126b |  |
| dre-miR-126b* |  |
| dre-miR-128 | yes |
| dre-miR-129 | yes |
| dre-miR-129* | yes |
| dre-miR-130a | yes |
| dre-miR-130b | yes |
| dre-miR-130c |  |
| dre-miR-132 | yes |
| dre-miR-132* | yes |
| dre-miR-133a | yes |
| dre-miR-133a* | yes |
| dre-miR-133b | yes |
| dre-miR-133c | yes |
| dre-miR-135a | yes |
| dre-miR-135b |  |
| dre-miR-135c | yes |
| dre-miR-137 | yes |
| dre-miR-138 | yes |
| dre-miR-1388 |  |
| dre-miR-1388* |  |
| dre-miR-139 | yes |
| dre-miR-140 | yes |
| dre-miR-140* | yes |
| dre-miR-141 | yes |
| dre-miR-142a-3p |  |
| dre-miR-142a-5p |  |
| dre-miR-142b-5p | yes |
| dre-miR-143 | yes |
| dre-miR-144 | yes |
| dre-miR-145 | yes |
| dre-miR-146a | yes |
| dre-miR-146b | yes |
| dre-miR-148 | yes |
| dre-miR-150 | yes |
| dre-miR-152 | yes |
| dre-miR-153a | yes |
| dre-miR-153b | yes |
| dre-miR-153c | yes |
| dre-miR-155 | yes |
| dre-miR-15a | yes |
| dre-miR-15a* | yes |
| dre-miR-15b | yes |
| dre-miR-16a |  |
| dre-miR-16b | yes |
| dre-miR-16c | yes |
| dre-miR-17a | yes |
| dre-miR-17a* | yes |
| dre-miR-181a | yes |
| dre-miR-181a* | yes |
| dre-miR-181b | yes |
| dre-miR-181c |  |
| dre-miR-182 | yes |
| dre-miR-183 | yes |
| dre-miR-184 | yes |
| dre-miR-187 | yes |
| dre-miR-18a | yes |
| dre-miR-18b | yes |
| dre-miR-18c | yes |
| dre-miR-190 | yes |
| dre-miR-190b | yes |
| dre-miR-192 | yes |
| dre-miR-193a | yes |
| dre-miR-193b | yes |
| dre-miR-194a | yes |
| dre-miR-196a | yes |
| dre-miR-196b | yes |
| dre-miR-199 | yes |
| dre-miR-199* | yes |
| dre-miR-19a |  |
| dre-miR-19a* | yes |
| dre-miR-19b | yes |
| dre-miR-19b* | yes |
| dre-miR-19c | yes |
| dre-miR-19d | yes |
| dre-miR-200a | yes |
| dre-miR-200b | yes |
| dre-miR-200c | yes |
| dre-miR-202* | yes |
| dre-miR-203a |  |
| dre-miR-203b | yes |
| dre-miR-203b* | yes |
| dre-miR-204 | yes |
| dre-miR-205 | yes |
| dre-miR-206 |  |
| dre-miR-20a |  |
| dre-miR-20a* | yes |
| dre-miR-20b | yes |
| dre-miR-21 | yes |
| dre-miR-210 | yes |
| dre-miR-210* | yes |
| dre-miR-212 |  |
| dre-miR-214 | yes |
| dre-miR-216a | yes |
| dre-miR-216b | yes |
| dre-miR-217 | yes |
| dre-miR-2184 |  |
| dre-miR-2187 | yes |
| dre-miR-2187* |  |
| dre-miR-2188 | yes |
| dre-miR-2188* | yes |
| dre-miR-218a | yes |
| dre-miR-218b | yes |
| dre-miR-219 | yes |
| dre-miR-221 | yes |
| dre-miR-222 | yes |
| dre-miR-223 | yes |
| dre-miR-22a | yes |
| dre-miR-22b | yes |
| dre-miR-23a | yes |
| dre-miR-23b | yes |
| dre-miR-24 | Yes |
| dre-miR-25 | yes |
| dre-miR-26a | yes |
| dre-miR-26b | yes |
| dre-miR-27a | yes |
| dre-miR-27b | yes |
| dre-miR-27c | yes |
| dre-miR-27d | yes |
| dre-miR-27e | yes |
| dre-miR-29a | yes |
| dre-miR-29b | yes |
| dre-miR-301a | yes |
| dre-miR-301b | yes |
| dre-miR-301c | yes |
| dre-miR-30b |  |
| dre-miR-30c | yes |
| dre-miR-30d | yes |
| dre-miR-30e | yes |
| dre-miR-30e* | yes |
| dre-miR-31 |  |
| dre-miR-338 | yes |
| dre-miR-34 | yes |
| dre-miR-34b | yes |
| dre-miR-34c | yes |
| dre-miR-363 | yes |
| dre-miR-365 | yes |
| dre-miR-375 | yes |
| dre-miR-429 | yes |
| dre-miR-429b |  |
| dre-miR-451 | yes |
| dre-miR-454a | yes |
| dre-miR-454b | yes |
| dre-miR-455 | yes |
| dre-miR-455b |  |
| dre-miR-456 | yes |
| dre-miR-457b | yes |
| dre-miR-458 | yes |
| dre-miR-459 |  |
| dre-miR-459* | yes |
| dre-miR-460-3p | yes |
| dre-miR-460-5p | yes |
| dre-miR-462 | yes |
| dre-miR-489 |  |
| dre-miR-499 | yes |
| dre-miR-722 | yes |
| dre-miR-723 | yes |
| dre-miR-724 | yes |
| dre-miR-725 | yes |
| dre-miR-726 | yes |
| dre-miR-727 | yes |
| dre-miR-727* | yes |
| dre-miR-728 | yes |
| dre-miR-730 | yes |
| dre-miR-731 | yes |
| dre-miR-734 | yes |
| dre-miR-736 | yes |
| dre-miR-7a | yes |
| dre-miR-7b |  |
| dre-miR-9 | yes |
| dre-miR-9* | yes |
| dre-miR-92a | yes |
| dre-miR-92b | yes |
| dre-miR-93 | yes |
| dre-miR-96 | yes |
| dre-miR-99 |  |
