# Supplementary material for: Global Analysis of the Small RNA Transcriptome in Different Ploidies and Genomic Combinations of a Vertebrate Complex – The Squalius alburnoides
Source: PLoS One. 2012 Jul 18;7(7):e41158. doi: 10.1371/journal.pone.0041158 (PMC3399795; doi:10.1371/journal.pone.0041158)
Supplement: Table S2 — Annotation of small RNA tags. (DOCX) [file pone.0041158.s003.docx]

|  | **PAA** | | **PA** | | **AA** | | **PP** | |
| --- | --- | --- | --- | --- | --- | --- | --- | --- |
| **Category** | **reads** | **%** | **reads** | **%** | **reads** | **%** | **reads** | **%** |
| **Total** | 43461092 | 100 | 43137518 | 100 | 44384528 | 100 | 42549707 | 100 |
| **miRNA** | 18666251 | 42.95 | 26938989 | 62.45 | 26168020 | 58.96 | 18814080 | 44.22 |
| **tRNA** | 2813046 | 6.47 | 1330317 | 3.08 | 2672865 | 6.02 | 4494841 | 10.56 |
| **rRNA** | 2535056 | 5.83 | 1687768 | 3.91 | 1661352 | 3.74 | 1707323 | 4.01 |
| **exon_sense** | 791073 | 1.82 | 459180 | 1.06 | 700137 | 1.58 | 1027195 | 2.41 |
| **exon_antisense** | 97637 | 0.22 | 70305 | 0.16 | 106634 | 0.24 | 240268 | 0.56 |
| **intron_sense** | 91026 | 0.21 | 67837 | 0.16 | 115119 | 0.26 | 114563 | 0.27 |
| **intron_antisense** | 61297 | 0.14 | 42862 | 0.10 | 79469 | 0.18 | 187842 | 0.44 |
| **snRNA** | 93142 | 0.21 | 59797 | 0.14 | 54724 | 0.12 | 128340 | 0.30 |
| **snoRNA** | 163141 | 0.38 | 99150 | 0.23 | 67818 | 0.15 | 91638 | 0.22 |
| **unann** | 18149423 | 41.76 | 12381313 | 28.70 | 12758390 | 28.75 | 15743617 | 37.00 |
