# Supplementary material for: Global Analysis of the Small RNA Transcriptome in Different Ploidies and Genomic Combinations of a Vertebrate Complex – The Squalius alburnoides
Source: PLoS One. 2012 Jul 18;7(7):e41158. doi: 10.1371/journal.pone.0041158 (PMC3399795; doi:10.1371/journal.pone.0041158)
Supplement: Table S3 — Differences in the expression levels of each miRNA for all the possible pairwise comparisons – PAA/PA, PAA/PP, PAA/AA, AA/PP, PA/PP, PA/AA. Fold differences are logarithmized and presented in an ascending order. In grey background are values higher than 1 and lower than −1. (DOCX) [file pone.0041158.s004.docx]

| **PAA/PA** | | **PAA/PP** | | **PAA/AA** | |  | **AA/PP** | | **PA/PP** | | **PA/AA** | |
| --- | --- | --- | --- | --- | --- | --- | --- | --- | --- | --- | --- | --- |
| -0,975243394 | **dre-miR-1** | -1,255566173 | **dre-miR-92b** | -2,763714804 | **dre-miR-202*** |  | -2,07076 | **dre-miR-222** | -1,94595521 | **dre-miR-722** | -3,511387488 | **dre-miR-202*** |
| -0,76208281 | **dre-miR-27d** | -1,216511971 | **dre-miR-139** | -1,581843506 | **dre-miR-107b** |  | -1,9392 | **dre-miR-150** | -1,77212162 | **dre-miR-146b** | -3,330770215 | **dre-miR-459** |
| -0,646942546 | **dre-miR-724** | -1,08834974 | **dre-miR-489** | -1,440041819 | **dre-miR-18c** |  | -1,91384 | **dre-miR-139** | -1,57364006 | **dre-miR-25** | -2,896917917 | **dre-miR-20b** |
| -0,632348756 | **dre-miR-133a*** | -0,996344359 | **dre-miR-129*** | -1,391926972 | **dre-miR-22a** |  | -1,62888 | **dre-miR-458** | -1,36566796 | **dre-miR-17a*** | -2,60651865 | **dre-miR-459*** |
| -0,596968853 | **dre-miR-34c** | -0,975407094 | **dre-miR-222** | -1,243651591 | **dre-miR-20b** |  | -1,43976 | **dre-miR-31** | -1,30909733 | **dre-miR-20b** | -2,217381456 | **dre-miR-375** |
| -0,586529273 | **dre-miR-27b** | -0,960536878 | **dre-miR-150** | -0,978636045 | **dre-miR-93** |  | -1,39294 | **dre-miR-132*** | -1,26185788 | **dre-miR-429b** | -2,1826515 | **dre-miR-122** |
| -0,503509355 | **dre-miR-499** | -0,944666769 | **dre-miR-722** | -0,948787859 | **dre-miR-122** |  | -1,37465 | **dre-miR-92b** | -1,25429626 | **dre-miR-19d** | -2,10225009 | **dre-miR-18c** |
| -0,455603589 | **dre-miR-133c** | -0,936802638 | **dre-miR-7a** | -0,924969057 | **dre-miR-726** |  | -1,3283 | **dre-miR-146b** | -1,25420253 | **dre-miR-31** | -1,898102768 | **dre-miR-107b** |
| -0,446347923 | **dre-miR-2187*** | -0,895422103 | **dre-miR-34c** | -0,844385887 | **dre-miR-203a** |  | -1,30509 | **dre-miR-130b** | -1,24812519 | **dre-miR-222** | -1,655609286 | **dre-miR-726** |
| -0,431073572 | **dre-miR-34b** | -0,876831895 | **dre-miR-25** | -0,830265124 | **dre-miR-724** |  | -1,29354 | **dre-miR-130a** | -1,18720869 | **dre-miR-726** | -1,374218122 | **dre-miR-19c** |
| -0,412074211 | **dre-miR-16b** | -0,862162784 | **dre-miR-203b*** | -0,766144125 | **dre-miR-107** |  | -1,28023 | **dre-miR-129*** | -1,18434985 | **dre-miR-429** | -1,367167301 | **dre-miR-19d** |
| -0,409140807 | **dre-miR-24** | -0,833754008 | **dre-miR-2187** | -0,672087037 | **dre-miR-103** |  | -1,26273 | **dre-let-7e** | -1,12850734 | **dre-miR-129*** | -1,336883067 | **dre-miR-194a** |
| -0,387486856 | **dre-miR-16a** | -0,807081383 | **dre-miR-17a*** | -0,589573296 | **dre-miR-19c** |  | -1,1982 | **dre-miR-7a** | -1,11647226 | **dre-miR-146a** | -1,279831016 | **dre-miR-22a** |
| -0,385261556 | **dre-miR-10d** | -0,800789952 | **dre-miR-128** | -0,572268997 | **dre-miR-2188*** |  | -1,17756 | **dre-miR-489** | -1,11552021 | **dre-miR-92b** | -1,122284576 | **dre-miR-93** |
| -0,379265295 | **dre-miR-181a** | -0,790560849 | **dre-miR-132*** | -0,54622627 | **dre-miR-203b** |  | -1,17698 | **dre-let-7g** | -1,10569859 | **dre-miR-139** | -1,110068472 | **dre-miR-199** |
| -0,372004042 | **dre-miR-125c** | -0,7391513 | **dre-miR-125a** | -0,541438097 | **dre-miR-206** |  | -1,1047 | **dre-miR-153b** | -1,08977973 | **dre-miR-7a** | -1,087996907 | **dre-miR-722** |
| -0,358493249 | **dre-miR-181a*** | -0,715148889 | **dre-miR-203b** | -0,502711311 | **dre-miR-203b*** |  | -1,10167 | **dre-miR-99** | -1,08797851 | **dre-miR-489** | -1,054881199 | **dre-miR-107** |
| -0,336620435 | **dre-miR-9** | -0,696750541 | **dre-miR-7b** | -0,484246521 | **dre-miR-15b** |  | -1,10055 | **dre-miR-92a** | -1,06773193 | **dre-miR-200c** | -1,009375975 | **dre-miR-25** |
| -0,328588424 | **dre-miR-23b** | -0,648700903 | **dre-miR-125c** | -0,463425827 | **dre-miR-16b** |  | -1,09662 | **dre-miR-2187** | -1,067279 | **dre-miR-92a** | -0,993033038 | **dre-miR-736** |
| -0,301427767 | **dre-miR-730** | -0,614153176 | **dre-miR-99** | -0,451653735 | **dre-miR-181b** |  | -1,07463 | **dre-let-7i** | -1,04905693 | **dre-miR-2187** | -0,966733002 | **dre-miR-1388*** |
| -0,275123114 | **dre-miR-133b** | -0,598908626 | **dre-miR-22a** | -0,440457054 | **dre-miR-19d** |  | -1,07273 | **dre-miR-7b** | -0,97853699 | **dre-miR-223** | -0,951628521 | **dre-miR-17a*** |
| -0,273846982 | **dre-miR-128** | -0,559295969 | **dre-miR-727** | -0,42807862 | **dre-miR-727** |  | -1,03896 | **dre-miR-301a** | -0,97104741 | **dre-miR-150** | -0,936378795 | **dre-miR-200b** |
| -0,224702269 | **dre-miR-181b** | -0,550697345 | **dre-miR-181b** | -0,413062282 | **dre-miR-1388*** |  | -0,9601 | **dre-miR-221** | -0,91970579 | **dre-miR-122** | -0,903055877 | **dre-miR-429b** |
| -0,219872435 | **dre-miR-137** | -0,546608544 | **dre-miR-456** | -0,39308432 | **dre-miR-17a*** |  | -0,95206 | **dre-miR-30b** | -0,89849674 | **dre-miR-194a** | -0,84274276 | **dre-miR-214** |
| -0,217853539 | **dre-miR-101a** | -0,544708528 | **dre-miR-132** | -0,392698117 | **dre-miR-142a-5p** |  | -0,94323 | **dre-miR-135b** | -0,88407069 | **dre-miR-214** | -0,809322496 | **dre-miR-429** |
| -0,216899932 | **dre-miR-734** | -0,542380647 | **dre-miR-203a** | -0,391874819 | **dre-miR-16c** |  | -0,93343 | **dre-let-7f** | -0,85017085 | **dre-miR-132*** | -0,784497374 | **dre-miR-155** |
| -0,205926374 | **dre-miR-125b** | -0,527815368 | **dre-miR-181a** | -0,368132614 | **dre-miR-214** |  | -0,92489 | **dre-miR-34c** | -0,84231618 | **dre-miR-210*** | -0,782856614 | **dre-miR-103** |
| -0,201212622 | **dre-miR-181c** | -0,494752722 | **dre-miR-221** | -0,344498995 | **dre-miR-199*** |  | -0,86039 | **dre-miR-129** | -0,7984889 | **dre-miR-203b*** | -0,762433896 | **dre-miR-190b** |
| -0,166527963 | **dre-miR-203a** | -0,487985277 | **dre-miR-125b** | -0,334645842 | **dre-miR-181a** |  | -0,85794 | **dre-miR-722** | -0,78884968 | **dre-miR-2188** | -0,729516412 | **dre-miR-192** |
| -0,164919478 | **dre-miR-218b** | -0,482907633 | **dre-miR-92a** | -0,312610184 | **dre-miR-25** |  | -0,80661 | **dre-miR-9*** | -0,78599251 | **dre-miR-456** | -0,69091289 | **dre-miR-1388** |
| -0,162937165 | **dre-miR-216a** | -0,472161489 | **dre-miR-187** | -0,277482408 | **dre-miR-2188** |  | -0,79412 | **dre-let-7h** | -0,75052054 | **dre-miR-192** | -0,677869244 | **dre-miR-363** |
| -0,151957724 | **dre-miR-135b** | -0,47078675 | **dre-miR-205** | -0,264254833 | **dre-miR-734** |  | -0,79243 | **dre-let-7j** | -0,74210047 | **dre-miR-19b** | -0,677851923 | **dre-miR-203a** |
| -0,143484399 | **dre-miR-125a** | -0,456526088 | **dre-miR-726** | -0,240561059 | **dre-miR-23b** |  | -0,78488 | **dre-miR-133c** | -0,7356485 | **dre-miR-145** | -0,676897704 | **dre-miR-146a** |
| -0,140082342 | **dre-miR-92b** | -0,455133646 | **dre-miR-734** | -0,236554215 | **dre-miR-128** |  | -0,77806 | **dre-miR-212** | -0,73440482 | **dre-miR-458** | -0,6719027 | **dre-miR-223** |
| -0,131242422 | **dre-miR-219** | -0,443330002 | **dre-miR-458** | -0,23457424 | **dre-miR-27d** |  | -0,75908 | **dre-miR-132** | -0,73398679 | **dre-miR-204** | -0,662933077 | **dre-miR-200a** |
| -0,124360572 | **dre-miR-20a*** | -0,423144838 | **dre-miR-15b** | -0,229245966 | **dre-miR-20a*** |  | -0,75324 | **dre-miR-451** | -0,73137219 | **dre-miR-155** | -0,662734362 | **dre-miR-2188** |
| -0,121944993 | **dre-miR-26b** | -0,409418171 | **dre-miR-214** | -0,209069213 | **dre-miR-2187*** |  | -0,75183 | **dre-miR-124** | -0,73135239 | **dre-miR-199** | -0,652559557 | **dre-miR-206** |
| -0,117116509 | **dre-let-7c** | -0,40917004 | **dre-miR-210*** | -0,201108669 | **dre-miR-10d** |  | -0,74205 | **dre-miR-205** | -0,72641753 | **dre-miR-184** | -0,616887974 | **dre-miR-2188*** |
| -0,112821458 | **dre-miR-132** | -0,403555353 | **dre-miR-2188** | -0,198487276 | **dre-miR-216a** |  | -0,73059 | **dre-miR-126*** | -0,68577845 | **dre-miR-205** | -0,610353842 | **dre-miR-142a-5p** |
| -0,112089956 | **dre-miR-22a** | -0,401199449 | **dre-let-7g** | -0,172603821 | **dre-miR-375** |  | -0,72964 | **dre-miR-26b** | -0,68184662 | **dre-miR-363** | -0,588541386 | **dre-miR-15b** |
| -0,110849756 | **dre-miR-139** | -0,393356566 | **dre-miR-137** | -0,165891221 | **dre-miR-23a** |  | -0,72662 | **dre-miR-26a** | -0,67894479 | **dre-miR-727** | -0,547685066 | **dre-miR-727** |
| -0,104087074 | **dre-miR-124** | -0,384774888 | **dre-miR-124** | -0,159450294 | **dre-miR-140*** |  | -0,70302 | **dre-miR-135c** | -0,67223317 | **dre-let-7g** | -0,475070295 | **dre-miR-203b** |
| -0,102544113 | **dre-miR-725** | -0,359467576 | **dre-miR-204** | -0,134298139 | **dre-miR-21** |  | -0,69893 | **dre-miR-137** | -0,66477748 | **dre-miR-19c** | -0,443806187 | **dre-miR-146b** |
| -0,101390937 | **dre-miR-22b** | -0,354374765 | **dre-miR-181c** | -0,125287513 | **dre-miR-187** |  | -0,69711 | **dre-miR-125a** | -0,64787187 | **dre-miR-7b** | -0,439531734 | **dre-miR-731** |
| -0,094982517 | **dre-miR-26a** | -0,327543633 | **dre-miR-19d** | -0,098942735 | **dre-miR-24** |  | -0,69595 | **dre-miR-143** | -0,64403529 | **dre-miR-203b** | -0,438995054 | **dre-miR-203b*** |
| -0,078552698 | **dre-miR-16c** | -0,302033096 | **dre-miR-30b** | -0,089978731 | **dre-miR-199** |  | -0,69311 | **dre-miR-204** | -0,63633141 | **dre-miR-731** | -0,39956801 | **dre-miR-200c** |
| -0,071149975 | **dre-miR-203b** | -0,29917524 | **dre-miR-135b** | -0,088489078 | **dre-miR-125b** |  | -0,69141 | **dre-miR-130c** | -0,63483569 | **dre-miR-30c** | -0,394227952 | **dre-miR-101b** |
| -0,063710256 | **dre-miR-203b*** | -0,28843714 | **dre-miR-20a*** | -0,086750845 | **dre-miR-722** |  | -0,68134 | **dre-miR-30c** | -0,6343818 | **dre-miR-130c** | -0,392321345 | **dre-miR-462** |
| -0,061153488 | **dre-miR-199*** | -0,283634997 | **dre-let-7b** | -0,071601494 | **dre-miR-210** |  | -0,66815 | **dre-miR-200c** | -0,63231898 | **dre-miR-30b** | -0,387860226 | **dre-miR-727*** |
| -0,052677201 | **dre-miR-15a*** | -0,283346104 | **dre-let-7j** | -0,069376142 | **dre-miR-27b** |  | -0,66686 | **dre-miR-145** | -0,59570327 | **dre-miR-125a** | -0,378454334 | **dre-miR-455** |
| -0,048915048 | **dre-miR-7b** | -0,277317196 | **dre-let-7d** | -0,055965569 | **dre-miR-727*** |  | -0,65441 | **dre-miR-184** | -0,58904417 | **dre-miR-99** | -0,374541415 | **dre-miR-210** |
| -0,025376859 | **dre-let-7d** | -0,276504924 | **dre-miR-16a** | -0,04702716 | **dre-miR-125c** |  | -0,6137 | **dre-miR-153c** | -0,58138124 | **dre-miR-107b** | -0,344028093 | **dre-miR-19a*** |
| -0,025145384 | **dre-miR-99** | -0,273792104 | **dre-let-7h** | -0,042065116 | **dre-miR-125a** |  | -0,6017 | **dre-miR-125c** | -0,57953386 | **dre-miR-212** | -0,324528054 | **dre-miR-23a** |
| -0,02446571 | **dre-miR-15a** | -0,270151985 | **dre-miR-22b** | -0,03629482 | **dre-miR-456** |  | -0,59549 | **dre-miR-100** | -0,57730213 | **dre-miR-365** | -0,320795794 | **dre-miR-140*** |
| -0,012196174 | **dre-miR-338** | -0,268870055 | **dre-miR-103** | -0,020484492 | **dre-miR-27e** |  | -0,56496 | **dre-miR-723** | -0,56277349 | **dre-miR-221** | -0,31865321 | **dre-miR-19b** |
| -0,002198878 | **dre-let-7b** | -0,265079607 | **dre-miR-107b** | 0,002236298 | **dre-let-7d** |  | -0,56426 | **dre-miR-128** | -0,56180152 | **dre-miR-727*** | -0,313316121 | **dre-miR-16c** |
| -0,000407606 | **dre-miR-489** | -0,258629697 | **dre-miR-212** | 0,004008961 | **dre-miR-455b** |  | -0,56425 | **dre-miR-25** | -0,55321675 | **dre-miR-187** | -0,29824461 | **dre-miR-210*** |
| 0,010474154 | **dre-miR-150** | -0,248050814 | **dre-miR-107** | 0,005558519 | **dre-miR-19a*** |  | -0,56041 | **dre-miR-19b*** | -0,53826646 | **dre-let-7j** | -0,283339507 | **dre-miR-199*** |
| 0,023892193 | **dre-miR-27a** | -0,248049831 | **dre-miR-23b** | 0,01910057 | **dre-miR-18b** |  | -0,55591 | **dre-miR-193b** | -0,53683026 | **dre-miR-107** | -0,275636414 | **dre-miR-456** |
| 0,033863459 | **dre-miR-21** | -0,229864485 | **dre-miR-727*** | 0,021589503 | **dre-miR-133a*** |  | -0,54636 | **dre-miR-22b** | -0,52748208 | **dre-miR-15b** | -0,226945466 | **dre-miR-181b** |
| 0,039381096 | **dre-miR-140** | -0,22247838 | **dre-let-7c** | 0,029439541 | **dre-miR-34c** |  | -0,54405 | **dre-miR-210*** | -0,52697934 | **dre-miR-128** | -0,206300397 | **dre-miR-187** |
| 0,039845905 | **dre-miR-27e** | -0,210427595 | **dre-miR-451** | 0,035633045 | **dre-miR-140** |  | -0,54251 | **dre-miR-138** | -0,52551905 | **dre-miR-200b** | -0,183316577 | **dre-miR-724** |
| 0,042130965 | **dre-miR-126** | -0,199316484 | **dre-miR-100** | 0,06052735 | **dre-miR-181c** |  | -0,53948 | **dre-miR-29a** | -0,48685504 | **dre-miR-22a** | -0,175520623 | **dre-miR-18b** |
| 0,044624978 | **dre-miR-2188*** | -0,159988858 | **dre-miR-16c** | 0,067171978 | **dre-let-7b** |  | -0,5339 | **dre-miR-365** | -0,48602957 | **dre-miR-143** | -0,168155597 | **dre-miR-21** |
| 0,050293226 | **dre-miR-126*** | -0,15583604 | **dre-miR-153b** | 0,085190096 | **dre-miR-181a*** |  | -0,51116 | **dre-miR-499** | -0,48229414 | **dre-miR-130b** | -0,147428975 | **dre-miR-144** |
| 0,055963574 | **dre-miR-455b** | -0,147497032 | **dre-miR-27d** | 0,086263613 | **dre-miR-10b** |  | -0,51034 | **dre-miR-456** | -0,44062601 | **dre-let-7h** | -0,104879394 | **dre-miR-20a*** |
| 0,059573622 | **dre-miR-132*** | -0,142076785 | **dre-miR-365** | 0,089186778 | **dre-miR-489** |  | -0,4819 | **dre-miR-193a** | -0,43192344 | **dre-miR-132** | -0,088451127 | **dre-miR-27c** |
| 0,065817492 | **dre-miR-29a** | -0,136399735 | **dre-miR-145** | 0,094366138 | **dre-miR-455** |  | -0,45109 | **dre-miR-16a** | -0,41960388 | **dre-miR-18c** | -0,080440661 | **dre-miR-218a** |
| 0,067984391 | **dre-miR-221** | -0,111335082 | **dre-let-7i** | 0,098889418 | **dre-miR-219** |  | -0,44707 | **dre-miR-30e*** | -0,40894195 | **dre-miR-135c** | -0,071986373 | **dre-miR-184** |
| 0,081018884 | **dre-miR-187** | -0,105306127 | **dre-miR-193b** | 0,102471789 | **dre-miR-200b** |  | -0,43956 | **dre-miR-146a** | -0,39418585 | **dre-let-7i** | -0,068775355 | **dre-miR-145** |
| 0,086031174 | **dre-miR-153b** | -0,104475481 | **dre-miR-24** | 0,118013121 | **dre-miR-462** |  | -0,42343 | **dre-miR-19b** | -0,37968201 | **dre-miR-103** | -0,060324396 | **dre-miR-27e** |
| 0,090831336 | **dre-miR-100** | -0,088209032 | **dre-miR-138** | 0,119061478 | **dre-miR-92b** |  | -0,41636 | **dre-miR-460-3p** | -0,37937843 | **dre-miR-451** | -0,052885043 | **dre-miR-152** |
| 0,10317329 | **dre-miR-153a** | -0,077875791 | **dre-miR-26a** | 0,125397368 | **dre-miR-730** |  | -0,41493 | **dre-miR-181c** | -0,37588906 | **dre-miR-203a** | -0,051948613 | **dre-miR-455b** |
| 0,104300865 | **dre-miR-15b** | -0,071218677 | **dre-miR-140** | 0,13485916 | **dre-miR-210*** |  | -0,41402 | **dre-miR-17a*** | -0,366951 | **dre-let-7e** | -0,051345615 | **dre-miR-16b** |
| 0,109966165 | **dre-let-7a** | -0,067788277 | **dre-miR-135c** | 0,147060045 | **dre-miR-1388** |  | -0,41075 | **dre-miR-29b** | -0,36141275 | **dre-miR-460-3p** | -0,047348901 | **dre-miR-734** |
| 0,110775577 | **dre-miR-103** | -0,035325043 | **dre-miR-26b** | 0,156065048 | **dre-miR-194a** |  | -0,4024 | **dre-miR-454a** | -0,35982678 | **dre-miR-135a** | -0,043382011 | **dre-miR-365** |
| 0,111127461 | **dre-miR-206** | -0,031905975 | **dre-let-7a** | 0,163065143 | **dre-let-7c** |  | -0,39952 | **dre-miR-125b** | -0,33304815 | **dre-miR-193b** | -0,041883243 | **dre-miR-10b** |
| 0,119612447 | **dre-miR-727** | -0,031856607 | **dre-miR-135a** | 0,163931691 | **dre-miR-152** |  | -0,38767 | **dre-miR-135a** | -0,32603145 | **dre-miR-181b** | -0,040860762 | **dre-miR-204** |
| 0,128152857 | **dre-miR-10b** | -0,020874779 | **dre-let-7e** | 0,166062098 | **dre-miR-218a** |  | -0,38557 | **dre-let-7c** | -0,3147782 | **dre-miR-130a** | -0,035544111 | **dre-miR-216a** |
| 0,132126607 | **dre-miR-129*** | 0,016977156 | **dre-miR-19b** | 0,174560246 | **dre-miR-16a** |  | -0,37681 | **dre-let-7a** | -0,29848962 | **dre-miR-34c** | -0,030374578 | **dre-miR-183** |
| 0,143654531 | **dre-miR-93** | 0,035696105 | **dre-miR-184** | 0,181570794 | **dre-miR-155** |  | -0,37501 | **dre-miR-429** | -0,29018419 | **dre-miR-100** | -0,024514911 | **dre-miR-10c** |
| 0,145674458 | **dre-miR-728** | 0,040440941 | **dre-miR-29a** | 0,214342445 | **dre-miR-132** |  | -0,3735 | **dre-miR-126b** | -0,28209528 | **dre-miR-125b** | -0,00374205 | **dre-miR-140** |
| 0,147843429 | **dre-miR-138** | 0,042451085 | **dre-miR-27b** | 0,232859764 | **dre-miR-190b** |  | -0,3735 | **dre-miR-126b*** | -0,28147249 | **dre-let-7b** | 0,025573534 | **dre-miR-34** |
| 0,149701384 | **dre-miR-126b** | 0,04434668 | **dre-miR-129** | 0,234045782 | **dre-miR-10c** |  | -0,37322 | **dre-miR-196b** | -0,28072419 | **dre-miR-124** | 0,027619157 | **dre-let-7d** |
| 0,149701384 | **dre-miR-126b*** | 0,053433935 | **dre-miR-146a** | 0,253169699 | **dre-miR-9** |  | -0,35948 | **dre-miR-203b*** | -0,27673323 | **dre-miR-125c** | 0,027861908 | **dre-miR-135a** |
| 0,152940717 | **dre-miR-7a** | 0,060986452 | **dre-miR-130c** | 0,261370237 | **dre-miR-7a** |  | -0,35878 | **dre-miR-429b** | -0,25197671 | **dre-let-7d** | 0,033285222 | **dre-miR-92a** |
| 0,15607517 | **dre-miR-153c** | 0,061978949 | **dre-miR-460-3p** | 0,262837698 | **dre-miR-2187** |  | -0,35813 | **dre-miR-141** | -0,24476562 | **dre-miR-141** | 0,037298767 | **dre-miR-128** |
| 0,158642833 | **dre-miR-23a** | 0,063379739 | **dre-miR-9*** | 0,267258069 | **dre-miR-34** |  | -0,3565 | **dre-miR-18a** | -0,24190359 | **dre-miR-153b** | 0,044625454 | **dre-miR-181a** |
| 0,159379522 | **dre-miR-9*** | 0,102635502 | **dre-miR-30c** | 0,271242757 | **dre-miR-205** |  | -0,35083 | **dre-let-7b** | -0,23827009 | **dre-miR-734** | 0,046522938 | **dre-miR-30c** |
| 0,159960773 | **dre-miR-133a** | 0,106285835 | **dre-miR-23a** | 0,27617916 | **dre-miR-22b** |  | -0,3469 | **dre-miR-187** | -0,23608884 | **dre-miR-138** | 0,047577156 | **dre-miR-2187** |
| 0,161351501 | **dre-miR-140*** | 0,114227421 | **dre-miR-130a** | 0,276363184 | **dre-miR-34b** |  | -0,30662 | **dre-miR-223** | -0,1735205 | **dre-miR-137** | 0,051622305 | **dre-miR-142a-3p** |
| 0,166797529 | **dre-let-7h** | 0,119909724 | **dre-miR-19c** | 0,283860574 | **dre-miR-129*** |  | -0,27958 | **dre-let-7d** | -0,16879742 | **dre-miR-22b** | 0,053921813 | **dre-miR-148** |
| 0,16711376 | **dre-let-7f** | 0,151583873 | **dre-miR-17a** | 0,295652298 | **dre-miR-728** |  | -0,273 | **dre-miR-27a** | -0,16411294 | **dre-miR-20a*** | 0,054964453 | **dre-miR-460-3p** |
| 0,168914465 | **dre-miR-451** | 0,16454406 | **dre-let-7f** | 0,305547671 | **dre-miR-137** |  | -0,24816 | **dre-miR-17a** | -0,16229077 | **dre-miR-190b** | 0,056293434 | **dre-miR-205** |
| 0,170509889 | **dre-miR-29b** | 0,178618763 | **dre-miR-730** | 0,3230008 | **dre-miR-27c** |  | -0,23302 | **dre-miR-20a** | -0,16066622 | **dre-miR-129** | 0,05704786 | **dre-miR-130c** |
| 0,1868203 | **dre-miR-454a** | 0,184319298 | **dre-miR-9** | 0,333616076 | **dre-miR-204** |  | -0,23277 | **dre-miR-30d** | -0,15319852 | **dre-miR-181c** | 0,063095383 | **dre-miR-217** |
| 0,194627194 | **dre-miR-18b** | 0,194429831 | **dre-miR-18b** | 0,334936015 | **dre-miR-133a** |  | -0,22327 | **dre-miR-153a** | -0,14922042 | **dre-miR-19a*** | 0,069376857 | **dre-let-7b** |
| 0,204976521 | **dre-miR-129** | 0,200408571 | **dre-miR-19a*** | 0,344876028 | **dre-let-7a** |  | -0,22117 | **dre-miR-454b** | -0,14858645 | **dre-miR-181a** | 0,088033365 | **dre-miR-23b** |
| 0,208064895 | **dre-miR-2184** | 0,206339046 | **dre-miR-30d** | 0,355789707 | **dre-miR-135a** |  | -0,19678 | **dre-miR-731** | -0,14725389 | **dre-miR-135b** | 0,089600385 | **dre-miR-489** |
| 0,214955323 | **dre-miR-205** | 0,214562024 | **dre-miR-196b** | 0,356511342 | **dre-miR-101b** |  | -0,19319 | **dre-miR-181a** | -0,14190851 | **dre-let-7a** | 0,098052987 | **dre-miR-19a** |
| 0,215266542 | **dre-miR-2187** | 0,215060781 | **dre-miR-18a** | 0,357267434 | **dre-miR-725** |  | -0,1909 | **dre-miR-734** | -0,14121656 | **dre-miR-17a** | 0,101425284 | **dre-miR-125a** |
| 0,216822734 | **dre-miR-152** | 0,229548845 | **dre-miR-429** | 0,362221066 | **dre-miR-144** |  | -0,18798 | **dre-miR-10a** | -0,11127773 | **dre-miR-30d** | 0,106964261 | **dre-miR-17a** |
| 0,217661726 | **dre-miR-142a-5p** | 0,234738352 | **dre-miR-155** | 0,367026719 | **dre-miR-124** |  | -0,17392 | **dre-miR-727*** | -0,11063615 | **dre-miR-140** | 0,108435521 | **dre-miR-7a** |
| 0,227228878 | **dre-miR-18a** | 0,242640048 | **dre-miR-16b** | 0,374874791 | **dre-miR-218b** |  | -0,16895 | **dre-miR-203b** | -0,10720275 | **dre-miR-30e*** | 0,113377546 | **dre-miR-141** |
| 0,227705644 | **dre-miR-193b** | 0,242646767 | **dre-miR-18c** | 0,375958381 | **dre-miR-7b** |  | -0,16606 | **dre-miR-133b** | -0,10539825 | **dre-let-7c** | 0,117443297 | **dre-miR-125b** |
| 0,239347595 | **dre-miR-456** | 0,244677212 | **dre-miR-126*** | 0,380368925 | **dre-miR-126** |  | -0,14893 | **dre-miR-19a** | -0,09603616 | **dre-miR-9*** | 0,121510475 | **dre-miR-30d** |
| 0,241690536 | **dre-miR-34** | 0,258397494 | **dre-miR-153a** | 0,391800962 | **dre-miR-365** |  | -0,14267 | **dre-miR-15a** | -0,08147253 | **dre-miR-16c** | 0,149983841 | **dre-miR-728** |
| 0,246508759 | **dre-miR-218a** | 0,263531856 | **dre-miR-454a** | 0,39614988 | **dre-miR-100** |  | -0,14161 | **dre-miR-190** | -0,06675186 | **dre-miR-27c** | 0,151739967 | **dre-miR133a** |
| 0,254883983 | **dre-let-7j** | 0,263849343 | **dre-miR-15a** | 0,399722317 | **dre-miR-17a** |  | -0,13124 | **dre-miR-727** | -0,06426468 | **dre-miR-196b** | 0,174981242 | **dre-miR-133a** |
| 0,258566693 | **dre-miR-10c** | 0,274865619 | **dre-miR-153c** | 0,406490615 | **dre-miR-15a** |  | -0,12841 | **dre-miR-301c** | -0,05912095 | **dre-miR-217** | 0,184158888 | **dre-miR-10d** |
| 0,264391621 | **dre-miR-460-5p** | 0,288779728 | **dre-miR-199** | 0,439084872 | **dre-miR-30d** |  | -0,1261 | **dre-miR-2188** | -0,05239337 | **dre-miR-23a** | 0,185577558 | **dre-miR-31** |
| 0,270997345 | **dre-let-7g** | 0,298304577 | **dre-miR-27a** | 0,440382038 | **dre-miR-19b** |  | -0,1222 | **dre-miR-217** | -0,05089425 | **dre-miR-19a** | 0,198076912 | **dre-miR-182** |
| 0,27268172 | **dre-miR-222** | 0,311283121 | **dre-miR-133b** | 0,45057628 | **dre-miR-193b** |  | -0,11328 | **dre-miR-30e** | -0,02541293 | **dre-miR-29a** | 0,198548652 | **dre-miR-212** |
| 0,272979356 | **dre-miR-301b** | 0,314200222 | **dre-miR-122** | 0,454277313 | **dre-miR-138** |  | -0,10688 | **dre-miR-140** | -0,01220447 | **dre-miR-18a** | 0,20993661 | **dre-miR-143** |
| 0,278790332 | **dre-miR-196b** | 0,317688233 | **dre-miR-130b** | 0,465322101 | **dre-miR-221** |  | -0,09907 | **dre-miR-181b** | -0,00260607 | **dre-let-7f** | 0,222876636 | **dre-miR-193b** |
| 0,281791601 | **dre-miR-142b-5p** | 0,327774157 | **dre-miR-210** | 0,477313636 | **dre-miR-133b** |  | -0,06887 | **dre-miR-9** | -0,00023374 | **dre-miR-18b** | 0,230137841 | **dre-miR-219** |
| 0,282814392 | **dre-let-7i** | 0,330130035 | **dre-miR-2188*** | 0,478313777 | **dre-miR-460-3p** |  | -0,0606 | **dre-miR-301b** | 0,001965577 | **dre-miR-301a** | 0,234915863 | **dre-let-7a** |
| 0,288743075 | **dre-miR-107** | 0,336864417 | **dre-miR-133a** | 0,481640604 | **dre-miR-153a** |  | -0,05922 | **dre-miR-20a*** | 0,017070352 | **dre-miR-26a** | 0,23728471 | **dre-miR-2187*** |
| 0,29103844 | **dre-miR-458** | 0,340971391 | **dre-miR-301a** | 0,487490066 | **dre-miR-99** |  | -0,04131 | **dre-miR-214** | 0,017577223 | **dre-miR-375** | 0,254180477 | **dre-let-7j** |
| 0,292764057 | **dre-miR-17a** | 0,34421137 | **dre-miR-20b** | 0,492966111 | **dre-miR-146a** |  | -0,02099 | **dre-miR-192** | 0,024791861 | **dre-miR-210** | 0,259149821 | **dre-miR-92b** |
| 0,298153153 | **dre-miR-193a** | 0,344742443 | **dre-miR-27c** | 0,496152494 | **dre-miR-459** |  | -0,00751 | **dre-miR-23b** | 0,070988546 | **dre-miR-101b** | 0,261745973 | **dre-miR-181c** |
| 0,301114895 | **dre-miR-723** | 0,361219072 | **dre-miR-30e*** | 0,503212749 | **dre-miR-338** |  | -0,00556 | **dre-miR-24** | 0,076675182 | **dre-miR-454a** | 0,268757275 | **dre-miR-460-5p** |
| 0,302945922 | **dre-miR-210** | 0,362519683 | **dre-miR-31** | 0,509058459 | **dre-let-7j** |  | -0,00396 | **dre-miR-363** | 0,080502218 | **dre-miR-23b** | 0,280187653 | **dre-let-7c** |
| 0,316265262 | **dre-miR-107b** | 0,371119626 | **dre-miR-429b** | 0,510493983 | **dre-miR-200a** |  | 0,001904 | **dre-miR-133a** | 0,086583575 | **dre-miR-26b** | 0,280320172 | **dre-miR-216b** |
| 0,317580397 | **dre-miR-30d** | 0,387854429 | **dre-miR-731** | 0,514273506 | **dre-miR-2184** |  | 0,021717 | **dre-miR-27c** | 0,090614072 | **dre-miR-723** | 0,29409796 | **dre-miR-135c** |
| 0,320867789 | **dre-miR-212** | 0,391765341 | **dre-miR-723** | 0,519410441 | **dre-miR-212** |  | 0,053143 | **dre-miR-155** | 0,091009671 | **dre-miR-20a** | 0,305324545 | **dre-miR-100** |
| 0,325409434 | **dre-miR-190** | 0,407971387 | **dre-miR-21** | 0,520301762 | **dre-let-7h** |  | 0,053197 | **dre-miR-730** | 0,110945557 | **dre-miR-16a** | 0,306214612 | **dre-miR-2184** |
| 0,3270816 | **dre-miR-20a** | 0,418127646 | **dre-miR-20a** | 0,53043104 | **dre-miR-145** |  | 0,061077 | **dre-miR-15b** | 0,118754075 | **dre-miR-153c** | 0,306439885 | **dre-miR-138** |
| 0,3279338 | **dre-miR-135a** | 0,430374981 | **dre-miR-200c** | 0,533142896 | **dre-miR-460-5p** |  | 0,087053 | **dre-miR-27d** | 0,127170286 | **dre-miR-459** | 0,308972508 | **dre-miR-196b** |
| 0,330249511 | **dre-miR-30b** | 0,44656744 | **dre-miR-27e** | 0,541529121 | **dre-miR-363** |  | 0,111803 | **dre-miR-27b** | 0,15518783 | **dre-miR-153a** | 0,310204073 | **dre-miR-24** |
| 0,331900658 | **dre-miR-727*** | 0,456035163 | **dre-miR-143** | 0,542788448 | **dre-miR-451** |  | 0,112889 | **dre-miR-19d** | 0,17686727 | **dre-miR-133a** | 0,315594606 | **dre-miR-301c** |
| 0,33896944 | **dre-miR-301a** | 0,461314474 | **dre-miR-181a*** | 0,547999844 | **dre-miR-1** |  | 0,136526 | **dre-miR-126** | 0,177706418 | **dre-miR-190** | 0,31933113 | **dre-miR-190** |
| 0,341117298 | **dre-miR-135c** | 0,461435547 | **dre-miR-133c** | 0,55638797 | **dre-miR-101a** |  | 0,171588 | **dre-miR-725** | 0,187166449 | **dre-miR-301c** | 0,319762166 | **dre-miR-30b** |
| 0,346039847 | **dre-let-7e** | 0,503152227 | **dre-miR-190** | 0,571275774 | **dre-miR-27a** |  | 0,175305 | **dre-miR-18b** | 0,194347612 | **dre-miR-126*** | 0,324043142 | **dre-miR-20a** |
| 0,349089958 | **dre-miR-30e** | 0,513373905 | **dre-miR-200b** | 0,571533576 | **dre-miR-18a** |  | 0,194826 | **dre-miR-19a*** | 0,219775397 | **dre-miR-144** | 0,324982883 | **dre-miR-125c** |
| 0,349592613 | **dre-miR-19a*** | 0,516919596 | **dre-miR-126** | 0,579895751 | **dre-miR-29a** |  | 0,231861 | **dre-miR-16c** | 0,27437601 | **dre-miR-27a** | 0,327169903 | **dre-miR-132** |
| 0,355111608 | **dre-miR-216b** | 0,528879521 | **dre-miR-725** | 0,584611735 | **dre-miR-731** |  | 0,245876 | **dre-miR-460-5p** | 0,277107318 | **dre-miR-1388** | 0,338243961 | **dre-miR-126** |
| 0,374482839 | **dre-miR-204** | 0,537594115 | **dre-miR-363** | 0,587756839 | **dre-miR-196b** |  | 0,245986 | **dre-miR-728** | 0,285468683 | **dre-miR-2188*** | 0,339886753 | **dre-miR-30e*** |
| 0,385257954 | **dre-miR-2188** | 0,541663248 | **dre-miR-728** | 0,602356076 | **dre-miR-132*** |  | 0,272153 | **dre-miR-23a** | 0,287557697 | **dre-miR-200a** | 0,344310698 | **dre-miR-18a** |
| 0,411457928 | **dre-miR-27c** | 0,552276716 | **dre-miR-218a** | 0,604533824 | **dre-miR-429** |  | 0,294888 | **dre-miR-142b-5p** | 0,288278679 | **dre-miR-15a** | 0,353510233 | **dre-let-7h** |
| 0,423355324 | **dre-miR-460-3p** | 0,557274584 | **dre-miR-140*** | 0,617614218 | **dre-miR-92a** |  | 0,301981 | **dre-miR-203a** | 0,304628952 | **dre-miR-24** | 0,373879983 | **dre-miR-451** |
| 0,428969242 | **dre-miR-130a** | 0,58299728 | **dre-miR-126b** | 0,635209257 | **dre-miR-135c** |  | 0,367222 | **dre-miR-144** | 0,305731583 | **dre-miR-218a** | 0,377576097 | **dre-miR-22b** |
| 0,43310977 | **dre-miR-210*** | 0,58299728 | **dre-miR-126b*** | 0,63542578 | **dre-miR-216b** |  | 0,3761 | **dre-miR-181a*** | 0,309848382 | **dre-miR-30e** | 0,378473315 | **dre-miR-153a** |
| 0,435188973 | **dre-miR-365** | 0,590142539 | **dre-miR-19a** | 0,644032458 | **dre-miR-135b** |  | 0,378734 | **dre-miR-199** | 0,320673649 | **dre-miR-301b** | 0,381289793 | **dre-miR-301b** |
| 0,453734538 | **dre-miR-301c** | 0,593689379 | **dre-miR-301b** | 0,644734563 | **dre-miR-190** |  | 0,38619 | **dre-miR-218a** | 0,374071553 | **dre-miR-21** | 0,391162511 | **dre-miR-96** |
| 0,468385447 | **dre-miR-30e*** | 0,594493753 | **dre-miR-194a** | 0,648717948 | **dre-miR-26a** |  | 0,399351 | **dre-miR-210** | 0,387209079 | **dre-miR-10c** | 0,397343711 | **dre-miR-221** |
| 0,472826472 | **dre-miR-455** | 0,613667479 | **dre-miR-223** | 0,650005676 | **dre-miR-30b** |  | 0,403192 | **dre-miR-103** | 0,389999895 | **dre-miR-193a** | 0,423144837 | **dre-miR-30e** |
| 0,474616146 | **dre-miR-214** | 0,621082033 | **dre-miR-724** | 0,651118742 | **dre-miR-20a** |  | 0,410878 | **dre-miR-200b** | 0,395886709 | **dre-miR-140*** | 0,424879429 | **dre-miR-7b** |
| 0,509656042 | **dre-miR-144** | 0,640937361 | **dre-miR-301c** | 0,654263148 | **dre-miR-301b** |  | 0,411742 | **dre-miR-10c** | 0,395952416 | **dre-miR-728** | 0,426831136 | **dre-miR-730** |
| 0,510340466 | **dre-miR-462** | 0,645812146 | **dre-miR-10c** | 0,665911041 | **dre-miR-454a** |  | 0,438404 | **dre-miR-194a** | 0,40668516 | **dre-miR-27e** | 0,430962326 | **dre-miR-15a** |
| 0,55367672 | **dre-miR-1388*** | 0,658974714 | **dre-miR-30e** | 0,690084886 | **dre-miR-184** |  | 0,45959 | **dre-miR-338** | 0,40830997 | **dre-miR-462** | 0,435652044 | **dre-miR-142b-5p** |
| 0,558550202 | **dre-miR-17a*** | 0,663519619 | **dre-miR-93** | 0,694293212 | **dre-miR-26b** |  | 0,465234 | **dre-miR-101b** | 0,433259522 | **dre-miR-126b** | 0,443689345 | **dre-miR-181a*** |
| 0,584334997 | **dre-miR-92a** | 0,669851405 | **dre-miR-29b** | 0,697305549 | **dre-miR-139** |  | 0,467027 | **dre-miR-27e** | 0,433259522 | **dre-miR-126b*** | 0,459817547 | **dre-miR-725** |
| 0,586991667 | **dre-miR-10a** | 0,688189422 | **dre-miR-193a** | 0,717437645 | **dre-miR-142b-5p** |  | 0,468418 | **dre-miR-726** | 0,441171873 | **dre-miR-455** | 0,471119794 | **dre-miR-124** |
| 0,599212396 | **dre-miR-145** | 0,698159051 | **dre-miR-499** | 0,729879259 | **dre-miR-429b** |  | 0,518069 | **dre-miR-107** | 0,474752256 | **dre-miR-126** | 0,479096742 | **dre-miR-454a** |
| 0,61257847 | **dre-miR-454b** | 0,727686655 | **dre-miR-146b** | 0,739047404 | **dre-miR-19a** |  | 0,542245 | **dre-miR-21** | 0,480010156 | **dre-miR-730** | 0,504767441 | **dre-let-7g** |
| 0,632298178 | **dre-miR-19b*** | 0,729467813 | **dre-miR-144** | 0,752373736 | **dre-miR-130c** |  | 0,55522 | **dre-miR-216b** | 0,485663615 | **dre-miR-19b*** | 0,51264145 | **dre-miR-99** |
| 0,641000418 | **dre-miR-19a** | 0,779043516 | **dre-miR-460-5p** | 0,769323143 | **dre-miR-301c** |  | 0,584032 | **dre-miR-218b** | 0,494218637 | **dre-miR-454b** | 0,51408426 | **dre-miR-29a** |
| 0,662214272 | **dre-miR-18c** | 0,783978997 | **dre-miR-2187*** | 0,772228794 | **dre-miR-30e** |  | 0,600161 | **dre-miR-190b** | 0,499305141 | **dre-miR-29b** | 0,515414924 | **dre-miR-338** |
| 0,695331877 | **dre-miR-130c** | 0,821770215 | **dre-miR-101b** | 0,775758785 | **dre-let-7g** |  | 0,605641 | **dre-miR-2184** | 0,514615521 | **dre-miR-460-5p** | 0,517159131 | **dre-miR-27b** |
| 0,696771791 | **dre-miR-25** | 0,83304526 | **dre-miR-190b** | 0,783951754 | **dre-miR-30c** |  | 0,622726 | **dre-miR-142a-3p** | 0,519828714 | **dre-miR-93** | 0,525426106 | **dre-miR-137** |
| 0,73064623 | **dre-miR-726** | 0,85849017 | **dre-miR-217** | 0,8082662 | **dre-miR-30e*** |  | 0,702931 | **dre-miR-148** | 0,520903358 | **dre-miR-9** | 0,527514571 | **dre-miR-27d** |
| 0,737434816 | **dre-miR-30c** | 0,869471468 | **dre-miR-142a-5p** | 0,841510726 | **dre-miR-148** |  | 0,706041 | **dre-miR-16b** | 0,53867961 | **dre-miR-10a** | 0,53980027 | **dre-miR-218b** |
| 0,747678686 | **dre-miR-202*** | 0,91403472 | **dre-miR-455** | 0,852247746 | **dre-miR-142a-3p** |  | 0,709458 | **dre-miR-19c** | 0,58636986 | **dre-miR-133b** | 0,542788454 | **dre-miR-132*** |
| 0,750745295 | **dre-miR-101b** | 0,91868681 | **dre-miR-462** | 0,869964326 | **dre-miR-9*** |  | 0,7167 | **dre-miR-140*** | 0,591539875 | **dre-miR-202*** | 0,547389581 | **dre-miR-27a** |
| 0,759041248 | **dre-miR-19b** | 0,92151696 | **dre-miR-199*** | 0,880661259 | **dre-miR-15a*** |  | 0,767029 | **dre-miR-34** | 0,614549404 | **dre-miR-27d** | 0,562053102 | **dre-miR-16a** |
| 0,76207726 | **dre-miR-184** | 0,958931163 | **dre-miR-218b** | 0,888540664 | **dre-miR-153c** |  | 0,792994 | **dre-miR-22a** | 0,628943983 | **dre-miR-27b** | 0,589796134 | **dre-miR-9** |
| 0,784650827 | **dre-miR-19c** | 0,962826823 | **dre-miR-338** | 0,904712488 | **dre-miR-129** |  | 0,800649 | **dre-miR-462** | 0,63138726 | **dre-miR-725** | 0,626414394 | **dre-miR-34c** |
| 0,787594914 | **dre-miR-148** | 0,964786832 | **dre-miR-455b** | 0,920259392 | **dre-miR-223** |  | 0,819644 | **dre-miR-455** | 0,651773368 | dre-miR-142a-5p | 0,653944259 | **dre-miR-133a*** |
| 0,799945998 | **dre-miR-130b** | 1,012349705 | **dre-miR-142b-5p** | 0,948841043 | **dre-miR-153b** |  | 0,902375 | **dre-miR-2188*** | 0,654677885 | **dre-miR-16b** | 0,655591953 | **dre-miR-723** |
| 0,800631442 | **dre-miR-142a-3p** | 1,034311504 | **dre-miR-34** | 0,956469721 | **dre-miR-126b** |  | 0,950509 | **dre-miR-200a** | 0,674330279 | **dre-miR-142a-3p** | 0,68046003 | **dre-let-7i** |
| 0,837978936 | **dre-miR-1388** | 1,075589696 | **dre-miR-192** | 0,956469721 | **dre-miR-126b*** |  | 0,960753 | **dre-miR-455b** | 0,73052173 | **dre-miR-142b-5p** | 0,699741967 | **dre-miR-129** |
| 0,917574749 | **dre-miR-217** | 1,105502517 | **dre-miR-216a** | 0,956700847 | **dre-miR-723** |  | 0,968038 | **dre-miR-1388** | 0,75683534 | **dre-miR-148** | 0,707442757 | **dre-miR-34b** |
| 0,926716248 | **dre-miR-19d** | 1,106833481 | **dre-miR-454b** | 0,963268421 | **dre-let-7i** |  | 0,993024 | **dre-miR-2187*** | 0,792584593 | **dre-miR-34** | 0,710590804 | **dre-miR-9*** |
| 0,942028357 | **dre-miR-143** | 1,115122628 | **dre-miR-1388** | 0,975243116 | **dre-miR-126*** |  | 1,097919 | **dre-miR-96** | 0,819771349 | **dre-miR-181a*** | 0,71541126 | **dre-miR-454b** |
| 0,966074169 | **dre-miR-155** | 1,117998167 | **dre-miR-19b*** | 0,978643098 | **dre-miR-150** |  | 1,12191 | **dre-miR-219** | 0,835522657 | **dre-miR-216b** | 0,726674497 | **dre-miR-10a** |
| 0,99529966 | **dre-miR-190b** | 1,119938866 | **dre-miR-2184** | 0,980664132 | **dre-miR-217** |  | 1,144042 | **dre-miR-34b** | 0,847542147 | **dre-miR-1388*** | 0,732471495 | **dre-miR-153c** |
| 1,001252062 | **dre-miR-722** | 1,125707652 | **dre-miR-10a** | 1,080574949 | **dre-miR-29b** |  | 1,146062 | **dre-miR-10b** | 0,908786883 | **dre-miR-455b** | 0,743706465 | **dre-miR-26a** |
| 1,020095741 | **dre-miR-199** | 1,156584111 | **dre-miR-141** | 1,095327391 | **dre-miR-222** |  | 1,20253 | **dre-miR-15a*** | 0,911837597 | **dre-miR-2184** | 0,75244275 | **dre-miR-133b** |
| 1,024149469 | **dre-miR-731** | 1,1623927 | **dre-miR-206** | 1,096551453 | **dre-miR-192** |  | 1,225901 | **dre-miR-101a** | 0,917002761 | **dre-miR-133c** | 0,77424751 | **dre-miR-101a** |
| 1,038856585 | **dre-miR-200b** | 1,190670639 | **dre-miR-216b** | 1,097948282 | **dre-let-7f** |  | 1,262145 | **dre-miR-142a-5p** | 0,974986623 | **dre-miR-338** | 0,795996183 | **dre-miR-135b** |
| 1,168843626 | **dre-miR-182** | 1,220823576 | **dre-miR-219** | 1,098496526 | **dre-miR-200c** |  | 1,262964 | **dre-miR-122** | 0,982634074 | **dre-miR-199*** | 0,806774337 | **dre-miR-126b** |
| 1,169869816 | **dre-miR-146a** | 1,232349734 | **dre-miR-10b** | 1,151958967 | **dre-miR-143** |  | 1,265991 | **dre-miR-199*** | 1,051228864 | **dre-miR-206** | 0,806774337 | **dre-miR-126b*** |
| 1,17343306 | **dre-miR-200a** | 1,339254935 | **dre-miR-202*** | 1,170064411 | **dre-miR-193a** |  | 1,303965 | **dre-miR-216a** | 1,104160503 | **dre-miR-10b** | 0,808161306 | **dre-miR-139** |
| 1,219404366 | **dre-miR-363** | 1,401255241 | **dre-miR-1388*** | 1,18552146 | **dre-miR-458** |  | 1,316739 | **dre-miR-107b** | 1,123814266 | **dre-miR-218b** | 0,816244205 | **dre-miR-26b** |
| 1,233869641 | **dre-miR-122** | 1,420430069 | **dre-miR-34b** | 1,209291837 | **dre-miR-499** |  | 1,348666 | **dre-miR-183** | 1,201632031 | **dre-miR-499** | 0,822651671 | **dre-miR-222** |
| 1,40131336 | **dre-miR-141** | 1,461027131 | **dre-miR-200a** | 1,231832817 | **dre-miR-459*** |  | 1,451323 | **dre-miR-724** | 1,230290546 | **dre-miR-2187*** | 0,822818616 | **dre-miR-130b** |
| 1,41386232 | **dre-miR-429** | 1,474998095 | **dre-miR-142a-3p** | 1,241829625 | **dre-let-7e** |  | 1,587838 | **dre-miR-20b** | 1,267988205 | **dre-miR-724** | 0,862815869 | **dre-miR-153b** |
| 1,456075583 | **dre-miR-183** | 1,544466628 | **dre-miR-148** | 1,246286902 | **dre-miR-133c** |  | 1,642131 | **dre-miR-93** | 1,268403307 | **dre-miR-216a** | 0,871917259 | **dre-miR-193a** |
| 1,492954116 | **dre-miR-194a** | 1,782313678 | **dre-miR-101a** | 1,313660164 | **dre-miR-10a** |  | 1,682664 | **dre-miR-18c** | 1,318274011 | **dre-miR-183** | 0,89448902 | **dre-miR-458** |
| 1,498070537 | **dre-miR-200c** | 2,062397233 | **dre-miR-375** | 1,32798373 | **dre-miR-454b** |  | 1,703806 | **dre-miR-206** | 1,352029623 | **dre-miR-219** | 0,895795778 | **dre-let-7e** |
| 1,592168092 | **dre-miR-223** | 2,083215468 | **dre-miR-15a*** | 1,366914538 | **dre-miR-182** |  | 1,814293 | **dre-miR-1388*** | 1,489064144 | **dre-miR-96** | 0,91007106 | **dre-miR-29b** |
| 1,61668584 | **dre-miR-31** | 2,108761839 | **dre-miR-10d** | 1,379903453 | **dre-miR-301a** |  | 2,181177 | **dre-miR-182** | 1,851467267 | **dre-miR-34b** | 0,924955891 | **dre-miR-126*** |
| 1,632941137 | **dre-miR-429b** | 2,35443094 | **dre-miR-152** | 1,407740581 | **dre-miR-130a** |  | 2,190475 | **dre-miR-152** | 2,000130844 | **dre-miR-101a** | 0,930840523 | **dre-let-7f** |
| 1,653272327 | **dre-miR-20b** | 2,774385968 | **dre-miR-183** | 1,425695005 | **dre-miR-183** |  | 2,234977 | **dre-miR-375** | 2,135856295 | **dre-miR-15a*** | 0,93334446 | **dre-miR-15a*** |
| 1,826073866 | **dre-miR-192** | 3,001128112 | **dre-miR-133a*** | 1,514684906 | **dre-miR-141** |  | 2,309846 | **dre-miR-10d** | 2,137571832 | **dre-miR-152** | 0,968174944 | **dre-miR-150** |
| 2,043751448 | **dre-miR-96** | 3,005436786 | **dre-miR-1** | 1,622758614 | **dre-miR-130b** |  | 2,457412 | **dre-miR-1** | 2,379235774 | **dre-miR-182** | 0,978777339 | **dre-miR-130a** |
| 2,044783636 | **dre-miR-375** | 3,532851966 | **dre-miR-96** | 1,678382089 | **dre-miR-19b*** |  | 2,979514 | **dre-miR-133a*** | 2,493987021 | **dre-miR-10d** | 1,040940014 | **dre-miR-301a** |
| 2,499771902 | **dre-miR-146b** | 3,548115775 | **dre-miR-182** | 1,802257397 | **dre-miR-31** |  | 3,457958 | **dre-miR-459** | 3,633440493 | **dre-miR-133a*** | 1,046089911 | **dre-miR-19b*** |
| 3,826928709 | **dre-miR-459** | 3,95413537 | **dre-miR-459** | 2,055959714 | **dre-miR-146b** |  | 4,102945 | **dre-miR-202*** | 3,980643806 | **dre-miR-1** | 1,523249239 | **dre-miR-1** |
| 3,838357468 | **dre-miR-459*** |  |  | 2,434907958 | **dre-miR-96** |  |  |  |  |  | 1,701896491 | **dre-miR-133c** |
|  |  |  |  |  |  |  |  |  |  |  | 1,712807193 | **dre-miR-499** |
