# Supplementary material for: Global Analysis of the Small RNA Transcriptome in Different Ploidies and Genomic Combinations of a Vertebrate Complex – The Squalius alburnoides
Source: PLoS One. 2012 Jul 18;7(7):e41158. doi: 10.1371/journal.pone.0041158 (PMC3399795; doi:10.1371/journal.pone.0041158)
Supplement: Table S4 — Differences between real expression values and expected values in case of additivity. Results are logarithmized log2 (real/expected)) and presented in an ascending order. In grey background are values higher than 1 and lower than −1. (DOCX) [file pone.0041158.s005.docx]

| **PAA** |  |  | **PA** |  |
| --- | --- | --- | --- | --- |
| -2,218101708 | **dre-miR-202*** | | -2,5831 | **dre-miR-202*** |
| -1,259176045 | **dre-miR-107b** | | -2,44661 | **dre-miR-459** |
| -1,171306651 | **dre-miR-22a** | | -2,30193 | **dre-miR-20b** |
| -1,062208323 | **dre-miR-18c** | | -1,67601 | **dre-miR-122** |
| -0,878990395 | **dre-miR-20b** | | -1,59645 | **dre-miR-459*** |
| -0,783769918 | **dre-miR-726** | | -1,57173 | **dre-miR-722** |
| -0,74934375 | **dre-miR-203a** | | -1,48575 | **dre-miR-375** |
| -0,635266703 | **dre-miR-122** | | -1,48414 | **dre-miR-18c** |
| -0,631824904 | **dre-miR-203b*** | | -1,43155 | **dre-miR-726** |
| -0,611910605 | **dre-miR-107** | | -1,37582 | **dre-miR-107b** |
| -0,606341891 | **dre-miR-93** | | -1,31067 | **dre-miR-25** |
| -0,603752297 | **dre-miR-203b** | | -1,30323 | **dre-miR-19d** |
| -0,548518948 | **dre-miR-103** | | -1,24784 | **dre-miR-146b** |
| -0,543787786 | **dre-miR-17a*** | | -1,16512 | **dre-miR-17a*** |
| -0,525344448 | **dre-miR-25** | | -1,1255 | **dre-miR-194a** |
| -0,495040338 | **dre-miR-92b** | | -1,08523 | **dre-miR-429b** |
| -0,485917244 | **dre-miR-724** | | -1,05376 | **dre-miR-19c** |
| -0,484388372 | **dre-miR-181b** | | -1,00064 | **dre-miR-429** |
| -0,472131503 | **dre-miR-727** | | -0,9282 | **dre-miR-22a** |
| -0,463047982 | **dre-miR-15b** | | -0,92436 | **dre-miR-199** |
| -0,449294448 | **dre-miR-128** | | -0,90505 | **dre-miR-146a** |
| -0,431844279 | **dre-miR-722** | | -0,85503 | **dre-miR-214** |
| -0,416868338 | **dre-miR-489** | | -0,82497 | **dre-miR-223** |
| -0,402650873 | **dre-miR-19d** | | -0,81017 | **dre-miR-107** |
| -0,400955853 | **dre-miR-181a** | | -0,76379 | **dre-miR-200c** |
| -0,388116582 | **dre-miR-19c** | | -0,74961 | **dre-miR-155** |
| -0,380957213 | **dre-miR-214** | | -0,73677 | **dre-miR-200b** |
| -0,34790315 | **dre-miR-34c** | | -0,73152 | **dre-miR-192** |
| -0,329730953 | **dre-miR-734** | | -0,71869 | **dre-miR-2188** |
| -0,327824223 | **dre-miR-2188*** | | -0,69908 | **dre-miR-31** |
| -0,319714568 | **dre-miR-2188** | | -0,67132 | **dre-miR-363** |
| -0,317448241 | **dre-miR-16c** | | -0,62155 | **dre-miR-203b*** |
| -0,312845379 | **dre-miR-125a** | | -0,61805 | **dre-miR-129*** |
| -0,277646172 | **dre-miR-129*** | | -0,6115 | **dre-miR-92a** |
| -0,275819128 | **dre-miR-125c** | | -0,60819 | **dre-miR-489** |
| -0,262771337 | **dre-miR-16b** | | -0,60633 | **dre-miR-727** |
| -0,255684857 | **dre-miR-7a** | | -0,6037 | **dre-miR-7a** |
| -0,2494926 | **dre-miR-187** | | -0,59453 | **dre-miR-2187** |
| -0,248187072 | **dre-miR-20a*** | | -0,58752 | **dre-miR-210*** |
| -0,245713181 | **dre-miR-139** | | -0,58655 | **dre-miR-103** |
| -0,241976548 | **dre-miR-23b** | | -0,5782 | **dre-miR-92b** |
| -0,233404752 | **dre-miR-125b** | | -0,55357 | **dre-miR-203b** |
| -0,22633284 | **dre-miR-456** | | -0,54976 | **dre-miR-15b** |
| -0,204998897 | **dre-miR-27d** | | -0,54856 | **dre-miR-222** |
| -0,200780878 | **dre-miR-2187** | | -0,54499 | **dre-miR-456** |
| -0,160768197 | **dre-miR-206** | | -0,53753 | **dre-miR-19b** |
| -0,115286695 | **dre-miR-727*** | | -0,53284 | **dre-miR-731** |
| -0,099703321 | **dre-miR-24** | | -0,52605 | **dre-miR-203a** |
| -0,096138119 | **dre-let-7d** | | -0,51392 | **dre-miR-93** |
| -0,090538512 | **dre-miR-181c** | | -0,48449 | **dre-miR-190b** |
| -0,084541847 | **dre-miR-132** | | -0,469 | **dre-miR-727*** |
| -0,07953237 | **dre-miR-23a** | | -0,43761 | **dre-miR-139** |
| -0,079318368 | **dre-miR-142a-5p** | | -0,43221 | **dre-miR-145** |
| -0,075355823 | **dre-miR-7b** | | -0,42779 | **dre-miR-184** |
| -0,069316161 | **dre-miR-210*** | | -0,42047 | **dre-miR-204** |
| -0,058565201 | **dre-let-7b** | | -0,3818 | **dre-miR-187** |
| -0,031913977 | **dre-miR-27b** | | -0,35378 | **dre-miR-205** |
| -0,030455308 | **dre-miR-199*** | | -0,32664 | **dre-miR-365** |
| -0,022109138 | **dre-miR-132*** | | -0,32582 | **dre-miR-30c** |
| -0,019855748 | **dre-miR-205** | | -0,32151 | **dre-miR-130c** |
| -0,01814327 | **dre-miR-1388*** | | -0,31838 | **dre-miR-1388*** |
| 0,000166657 | **dre-miR-140** | | -0,30779 | **dre-miR-132*** |
| 0,008902421 | **dre-miR-16a** | | -0,29768 | **dre-miR-150** |
| 0,018993384 | **dre-miR-150** | | -0,28066 | **dre-miR-125a** |
| 0,021256297 | **dre-miR-99** | | -0,27758 | **dre-miR-1388** |
| 0,023685496 | **dre-let-7c** | | -0,26885 | **dre-miR-181b** |
| 0,026163093 | **dre-miR-21** | | -0,264 | **dre-miR-128** |
| 0,026827196 | **dre-miR-199** | | -0,25556 | **dre-miR-200a** |
| 0,03395222 | **dre-miR-137** | | -0,24126 | **dre-miR-19a*** |
| 0,043701749 | **dre-miR-140*** | | -0,23418 | **dre-miR-212** |
| 0,047815277 | **dre-miR-222** | | -0,22613 | **dre-miR-2188*** |
| 0,050910051 | **dre-miR-210** | | -0,22537 | **dre-miR-30b** |
| 0,054069795 | **dre-miR-2187*** | | -0,20098 | **dre-miR-7b** |
| 0,064628494 | **dre-miR-204** | | -0,19338 | **dre-miR-16c** |
| 0,068808855 | **dre-miR-19a*** | | -0,19262 | **dre-let-7g** |
| 0,070705018 | **dre-miR-221** | | -0,18764 | **dre-let-7j** |
| 0,071031605 | **dre-miR-22b** | | -0,18618 | **dre-miR-23a** |
| 0,071464453 | **dre-miR-124** | | -0,17989 | **dre-miR-210** |
| 0,076379261 | **dre-miR-18b** | | -0,1715 | **dre-miR-101b** |
| 0,12034469 | **dre-miR-27e** | | -0,17143 | **dre-miR-143** |
| 0,122033105 | **dre-miR-216a** | | -0,17062 | **dre-miR-135a** |
| 0,144034369 | **dre-miR-730** | | -0,15987 | **dre-miR-460-3p** |
| 0,151961377 | **dre-miR-92a** | | -0,1531 | **dre-miR-221** |
| 0,170035819 | **dre-miR-100** | | -0,13752 | **dre-miR-734** |
| 0,191902457 | **dre-miR-365** | | -0,13291 | **dre-miR-99** |
| 0,194773271 | **dre-let-7j** |  | -0,13082 | **dre-miR-458** |
| 0,200190263 | **dre-miR-155** | | -0,12629 | **dre-miR-20a*** |
| 0,201262812 | **dre-miR-181a*** | | -0,11054 | **dre-let-7d** |
| 0,205231444 | **dre-let-7h** | | -0,10833 | **dre-let-7b** |
| 0,208956208 | **dre-let-7a** | | -0,10395 | **dre-miR-142a-5p** |
| 0,211781469 | **dre-miR-212** | | -0,09358 | **dre-miR-132** |
| 0,215577798 | **dre-miR-135a** | | -0,09165 | **dre-miR-135c** |
| 0,228147533 | **dre-miR-200b** | | -0,08939 | **dre-let-7h** |
| 0,230908247 | **dre-miR-9** | | -0,08778 | **dre-miR-125b** |
| 0,24138514 | **dre-miR-193b** | | -0,0819 | **dre-miR-18b** |
| 0,246578725 | **dre-miR-451** | | -0,07344 | **dre-miR-193b** |
| 0,247084541 | **dre-miR-10d** | | -0,06909 | **dre-miR-27c** |
| 0,25075312 | **dre-miR-138** | | -0,06842 | **dre-miR-141** |
| 0,257716617 | **dre-miR-135b** | | -0,04969 | **dre-miR-140** |
| 0,259349724 | **dre-miR-30b** | | -0,04677 | **dre-miR-181a** |
| 0,260599664 | **dre-miR-455b** | | -0,04321 | **dre-miR-451** |
| 0,268553614 | **dre-miR-375** | | -0,03789 | **dre-miR-462** |
| 0,270010858 | **dre-let-7g** | | -0,02944 | **dre-miR-206** |
| 0,273133744 | **dre-miR-145** | | -0,01712 | **dre-miR-455** |
| 0,284938258 | **dre-miR-218a** | | -0,0147 | **dre-miR-100** |
| 0,285904879 | **dre-miR-19b** | | -0,01404 | **dre-miR-17a** |
| 0,28922334 | **dre-miR-194a** | | 0,001212 | **dre-miR-125c** |
| 0,313148467 | **dre-miR-17a** | | 0,002408 | **dre-miR-140*** |
| 0,320921856 | **dre-miR-455** | | 0,008849 | **dre-miR-30d** |
| 0,326673956 | **dre-miR-460-3p** | | 0,009174 | **dre-miR-217** |
| 0,331310627 | **dre-miR-27c** | | 0,018084 | **dre-miR-138** |
| 0,331985605 | **dre-miR-146a** | | 0,030125 | **dre-miR-19a** |
| 0,336668097 | **dre-miR-133a** | | 0,033259 | **dre-miR-144** |
| 0,340347129 | **dre-miR-462** | | 0,035355 | **dre-miR-130b** |
| 0,358234558 | **dre-miR-30d** | | 0,042583 | **dre-let-7a** |
| 0,35837953 | **dre-miR-15a** | | 0,047735 | **dre-miR-181c** |
| 0,359958037 | **dre-miR-10c** | | 0,053311 | **dre-let-7i** |
| 0,361776357 | **dre-miR-135c** | | 0,054923 | **dre-miR-124** |
| 0,364636843 | **dre-miR-26a** | | 0,082894 | **dre-let-7c** |
| 0,37428873 | **dre-miR-728** | | 0,08646 | **dre-miR-21** |
| 0,377649781 | **dre-miR-29a** | | 0,086938 | **dre-miR-22b** |
| 0,378904257 | **dre-miR-10b** | | 0,092804 | **dre-miR-23b** |
| 0,387040812 | **dre-miR-219** | | 0,099153 | **dre-miR-34c** |
| 0,404304614 | **dre-miR-153a** | | 0,107403 | **dre-miR-30e*** |
| 0,405139179 | **dre-miR-1388** | | 0,10851 | **dre-miR-218a** |
| 0,407927316 | **dre-miR-190b** | | 0,118667 | **dre-miR-196b** |
| 0,408840328 | **dre-miR-26b** | | 0,138383 | **dre-let-7e** |
| 0,413402632 | **dre-miR-725** | | 0,142217 | **dre-miR-137** |
| 0,42082906 | **dre-miR-133b** | | 0,163155 | **dre-miR-27e** |
| 0,422306418 | **dre-miR-458** | | 0,163426 | **dre-miR-18a** |
| 0,425617694 | **dre-miR-126** | | 0,17547 | **dre-miR-10c** |
| 0,438286766 | **dre-miR-184** | | 0,18447 | **dre-miR-133a** |
| 0,443584803 | **dre-miR-18a** | | 0,199537 | **dre-miR-130a** |
| 0,453249893 | **dre-miR-196b** | | 0,211249 | **dre-miR-20a** |
| 0,469322058 | **dre-miR-429** | | 0,214429 | **dre-miR-129** |
| 0,475387186 | **dre-miR-27a** | | 0,215193 | **dre-miR-153b** |
| 0,47581552 | **dre-miR-144** | | 0,224197 | **dre-miR-199*** |
| 0,481034523 | **dre-miR-153b** | | 0,227527 | **dre-miR-29a** |
| 0,48201325 | **dre-miR-34** | | 0,255252 | **dre-miR-190** |
| 0,484145106 | **dre-miR-130c** | | 0,256683 | **dre-miR-135b** |
| 0,496863259 | **dre-miR-101b** | | 0,258429 | **dre-miR-301c** |
| 0,510981738 | **dre-let-7i** |  | 0,259753 | **dre-miR-9*** |
| 0,516993966 | **dre-miR-731** | | 0,267811 | **dre-miR-16b** |
| 0,519810769 | **dre-miR-133a*** | | 0,270943 | **dre-miR-153a** |
| 0,519847452 | **dre-miR-454a** | | 0,272234 | **dre-miR-454a** |
| 0,520218734 | **dre-miR-30c** | | 0,276407 | **dre-miR-728** |
| 0,541303041 | **dre-miR-363** | | 0,315956 | **dre-miR-24** |
| 0,54591859 | **dre-miR-218b** | | 0,327248 | **dre-miR-16a** |
| 0,549073476 | **dre-miR-9*** | | 0,338505 | **dre-miR-142a-3p** |
| 0,558452557 | **dre-miR-129** | | 0,343286 | **dre-miR-26a** |
| 0,568630844 | **dre-miR-34b** | | 0,353872 | **dre-miR-723** |
| 0,570177528 | **dre-miR-20a** | | 0,358915 | **dre-miR-455b** |
| 0,597000523 | **dre-miR-190** | | 0,359176 | **dre-miR-301b** |
| 0,600747939 | **dre-miR-152** | | 0,366327 | **dre-miR-15a** |
| 0,601036343 | **dre-miR-429b** | | 0,367636 | **dre-miR-34** |
| 0,611746556 | **dre-miR-460-5p** | | 0,371894 | **dre-miR-148** |
| 0,634847672 | **dre-miR-301b** | | 0,37387 | **dre-miR-30e** |
| 0,642059326 | **dre-miR-338** | | 0,376304 | **dre-miR-724** |
| 0,644240455 | **dre-miR-30e*** | | 0,39513 | **dre-miR-460-5p** |
| 0,654552662 | **dre-miR-153c** | | 0,397974 | **dre-let-7f** |
| 0,688702456 | **dre-miR-19a** | | 0,401448 | **dre-miR-153c** |
| 0,6893601 | **dre-miR-126*** | | 0,412836 | **dre-miR-27a** |
| 0,689903205 | **dre-let-7e** | | 0,413501 | **dre-miR-126** |
| 0,690700455 | **dre-miR-2184** | | 0,413936 | **dre-miR-26b** |
| 0,716434625 | **dre-let-7f** | | 0,429331 | **dre-miR-10b** |
| 0,726272935 | **dre-miR-301c** | | 0,437898 | **dre-miR-301a** |
| 0,734515295 | **dre-miR-30e** | | 0,461749 | **dre-miR-730** |
| 0,743662765 | **dre-miR-723** | | 0,483058 | **dre-miR-216a** |
| 0,764981321 | **dre-miR-200a** | | 0,500991 | **dre-miR-183** |
| 0,799197554 | **dre-miR-216b** | | 0,522056 | **dre-miR-126*** |
| 0,81042641 | **dre-miR-142b-5p** | | 0,540211 | **dre-miR-216b** |
| 0,811599643 | **dre-miR-223** | | 0,551689 | **dre-miR-725** |
| 0,821853504 | **dre-miR-126b** | | 0,563447 | **dre-miR-9** |
| 0,821853504 | **dre-miR-126b*** | | 0,578967 | **dre-miR-27d** |
| 0,838913315 | **dre-miR-130a** | | 0,580573 | **dre-miR-27b** |
| 0,840627213 | **dre-miR-200c** | | 0,584266 | **dre-miR-142b-5p** |
| 0,863439174 | **dre-miR-101a** | | 0,586337 | **dre-miR-2184** |
| 0,881699601 | **dre-miR-143** | | 0,609009 | **dre-miR-454b** |
| 0,931181112 | **dre-miR-29b** | | 0,616312 | **dre-miR-126b** |
| 0,935506168 | **dre-miR-133c** | | 0,616312 | **dre-miR-126b*** |
| 0,939809118 | **dre-miR-217** | | 0,619222 | **dre-miR-193a** |
| 0,945822399 | **dre-miR-301a** | | 0,628252 | **dre-miR-181a*** |
| 0,99180219 | **dre-miR-193a** | | 0,638063 | **dre-miR-10a** |
| 1,00912488 | **dre-miR-1** | | 0,659036 | **dre-miR-2187*** |
| 1,018904599 | **dre-miR-459** | | 0,670839 | **dre-miR-152** |
| 1,018912648 | **dre-miR-499** | | 0,675476 | **dre-miR-133b** |
| 1,032888125 | **dre-miR-142a-3p** | | 0,693781 | **dre-miR-219** |
| 1,041433841 | **dre-miR-148** | | 0,698448 | **dre-miR-29b** |
| 1,047549374 | **dre-miR-130b** | | 0,735763 | **dre-miR-338** |
| 1,090608974 | **dre-miR-192** | | 0,747087 | **dre-miR-19b*** |
| 1,151002766 | **dre-miR-31** | | 0,811304 | **dre-miR-218b** |
| 1,183561536 | **dre-miR-15a*** | | 0,847199 | **dre-miR-96** |
| 1,249248085 | **dre-miR-10a** | | 0,920123 | **dre-miR-182** |
| 1,251419769 | **dre-miR-454b** | | 0,928656 | **dre-miR-10d** |
| 1,386099681 | **dre-miR-141** | | 1,178027 | **dre-miR-34b** |
| 1,467279851 | **dre-miR-19b*** | | 1,264859 | **dre-miR-133c** |
| 1,467868733 | **dre-miR-146b** | | 1,269876 | **dre-miR-101a** |
| 1,753660031 | **dre-miR-183** | | 1,421923 | **dre-miR-15a*** |
| 1,802808407 | **dre-miR-182** | | 1,442976 | **dre-miR-499** |
| 1,818912622 | **dre-miR-459*** | | 1,491451 | **dre-miR-133a*** |
| 2,71853929 | **dre-miR-96** | | 2,291525 | **dre-miR-1** |
